# A Growth-Promoting Bacteria, *Paenibacillus yonginensis* DCY84^T^ Enhanced Salt Stress Tolerance by Activating Defense-Related Systems in *Panax ginseng*

**DOI:** 10.3389/fpls.2018.00813

**Published:** 2018-07-23

**Authors:** Johan Sukweenadhi, Sri R. Balusamy, Yeon-Ju Kim, Choong H. Lee, Yu-Jin Kim, Sung C. Koh, Deok C. Yang

**Affiliations:** ^1^Graduate School of Biotechnology, Kyung Hee University, Yongin, South Korea; ^2^Faculty of Biotechnology, University of Surabaya, Surabaya, Indonesia; ^3^Department of Food Science and Biotechnology, Sejong University, Seoul, South Korea; ^4^Department of Oriental Medicinal Biotechnology, College of Life Science, Kyung Hee University, Yongin, South Korea; ^5^Department of Environmental Engineering, Korea Maritime and Ocean University, Busan, South Korea; ^6^Department of Bioscience and Biotechnology, Konkuk University, Seoul, South Korea

**Keywords:** *Panax ginseng* Meyer, plant growth promoting bacteria, salinity stress, *Paenibacillus yonginensis*, abscisic acid

## Abstract

*Panax ginseng* (C.A. Mayer) is a well-known medicinal plant used in traditional medicine in Korea that experiences serious salinity stress related to weather changes or incorrect fertilizer application. In ginseng, the use of *Paenibacillus yonginensis* DCY84^T^ to improve salt stress tolerance has not been thoroughly explored. Therefore, we studied the role of *P. yonginensis* DCY84^T^ under short-term and long-term salinity stress conditions in a controlled environment. *In vitro* testing of DCY84^T^ revealed high indole acetic acid (IAA) production, siderophore formation, phosphate solubilization and anti-bacterial activity. We determined that 10-min dip in 10^10^ CFU/ml DCY84^T^ was sufficient to protect ginseng against short-term salinity stress (osmotic stress) upon exposure to 300 mM NaCl treatment by enhancing nutrient availability, synthesizing hydrolyzing enzymes and inducing osmolyte production. Upon exposure to salinity stress (oxidative and ionic stress), strain DCY84^T^-primed ginseng seedlings were protected by the induction of defense-related systems such as ion transport, ROS scavenging enzymes, proline content, total sugars, and ABA biosynthetic genes, as well as genes involved in root hair formation. Additionally, ginseng primed with DCY84^T^ and exposed to 300 mM NaCl showed the same metabolite profile as control ginseng plants, suggesting that DCY84^T^ effectively reduced salt stress. These results indicated that DCY84^T^ can be widely used as a microbial inoculant to protect ginseng plants against salinity stress conditions.

## Introduction

Salinity is a major threat to plant and environmental resources worldwide. Other factors that increase salinity include poor water management on cultivated lands, high evaporation with low precipitation, and poor cultural practices, such as improper fertilizer addition (Egamberdieva et al., 2007). Recent research in plant physiology has included omics-driven studies, bioinformatics, and new analytical techniques in fields such as metabolomics (Negrão et al., 2017). Generally, plants respond to salinity in two major phases: the shoot ion-dependent response that occurs between minutes to days after exposure; and the ion-dependent response that occurs over longer period (usually days to weeks), ultimately resulting in reduced yield and plant senescence. Plant growth and its productivity are impeded by salinity through interference with normal physiological and metabolic processes (Annunziata et al., 2017). Thus, further research is being conducted to understand plant responses to salinity and find new ways to improve salinity tolerance (Munns and Tester, 2008).

Plant species have individual sensitivity levels to salinity. Most plants (glycophytes) are very sensitive to soil salinity, but some plants (halophytes) are salt-tolerant. Halophytes have evolved many tolerance mechanisms to overcome adverse salinity conditions and to maintain their physiology and growth processes (Ahanger et al., 2014), including ion pumps (Hu and Schmidhalter, 2005), abscisic acid (ABA) (Zhu, 2002), osmoprotectants (Chinnusamy et al., 2005), and ROS scavenging (Vinocur and Altman, 2005). Understanding plant defenses against salinity stress and developing salinity-tolerant plants are crucial to solving the ongoing problem of soil salinity.

Plant growth-promoting bacteria (PGPB) form symbiotic relationships with plants that can benefit the plant (Forchetti et al., 2007; Diaz-Zorita and Fernandez-Canigia, 2009). PGPB are soil bacteria that facilitate plant growth and often colonize the rhizosphere; they can indirectly activate various antioxidant enzymes such as superoxide dismutase (SOD), peroxidase and catalase, which scavenge excess ROS and safeguard plants from salt toxicity (Jha and Subramanian, 2014; Islam et al., 2015). There are various mechanisms for plant growth promotion by PGPR including acquisition of nutrients, phosphorus (P) solubilization, siderophore production, fixation of atmospheric nitrogen (N), phosphorus (P) solubilization, hydrocyanic (HCN) production, regulation of plant hormones and defensive action against biotic pathogens. N fixation, P availability and hormonal response are directly involved in plant growth promotion; other mechanisms contribute to plant growth indirectly (Gontia et al., 2011; Bhattacharyya and Jha, 2012; Glick, 2012; Estrada et al., 2013; Vacheron et al., 2013). Plants that are inoculated with PGPR modulate root architecture due to increased indole-3-acetic acid (IAA) level, which allows plants to uptake more nutrients under salinity stress conditions (Vacheron et al., 2013; Goswami et al., 2014). Another report demonstrated that rhizobacteria release volatile compounds that increase the life span of plants under severe drought stress (Timmusk et al., 2014). Recently, Rolli et al. (2015) studied eight osmotolerant bacterial isolates that showed plant growth-promoting properties and improved grapevine growth under drought stress. Moreover, PGPR can serve as a bio-fertilizer or phyto-stimulator to maintain soil fertility, therefore providing a promising alternative approach to chemical fertilizers and pesticides in agricultural practices (Majeed et al., 2015).

*Panax ginseng* Meyer is a well-recognized medicinal plant in east Asia, including Korea, China and Japan. Its long cultivation period, usually 4–6 years, makes this valuable medicinal plant vulnerable to various environmental conditions, one of which is salinity (Egamberdieva and Da Silva, 2015). Several methods such as traditional breeding and genetic engineering have been implemented to improve ginseng plant salinity tolerance; however, little improvement has been observed (Krishna et al., 2015). PGPR is a highly effective and eco-friendly approach to improving salinity stress. Moreover, several reports have also shown that PGPR could effectively improve plant growth and prevent plants from various forms of environmental stress (Basillo et al., 2004; Mayak et al., 2004; Yuwono et al., 2005; Islam et al., 2015; Majeed et al., 2015; Rolli et al., 2015; Timmusk et al., 2015; Zahid et al., 2015). Our previous report suggested that DCY84^T^ could be a promising PGPB isolate for protection of *Arabidopsis* against salt stress (Sukweenadhi et al., 2015). Therefore, we attempted to study the role of PGPR in controlling salinity stress in ginseng. Several bacteria were isolated from ginseng soil and tested for plant growth-promoting activity and among them we chose DCY84^T^ *Paenibacillus yonginensis* because its full genome sequence had been reported to have several plant growth-promoting genes. In the present study, we used strain DCY84^T^ to increase salinity stress tolerance in ginseng seedlings in a controlled environment. To the best of our knowledge, this is the first study to show that DCY84^T^ can regulate salt tolerance defensive mechanisms in plants.

## Materials and methods

### Plant material and culture conditions

We obtained 2- and 4-year-old *P. ginseng* root seedlings from the Ginseng Resource Bank, Kyung Hee University. As the growth medium, we prepared artificial soil by mixing vermiculite, perlite, and peat moss at a 3:1:1 volume ratio. The mixed soil was autoclaved at 121°C for 1 h and then air-dried. The sterilization step was repeated twice on different days. Before culturing the roots, we added sterilized tap water to the mixed soil in a 25% *v/v* ratio. Later, we used the prepared soil to fill trays or pots for growing the ginseng. Each tray or pot was placed in an open cabinet with 60 × 100 cm dimension (0.6 m^2^) inside a closed room. We adjusted the photoperiod to a 16:8-h light:dark cycle using lamps (Philips TLD-RS-FLR32SSEX-D 865K) equal to 9500 lux for each covered area. The temperature was controlled at 25 ± 2°C, and the moisture level was maintained at 60 ± 5%. Sterilized tap water was sprayed onto the soil surface daily, and watering was done using sterilized tap water once a week from a plate beneath the tray/pot.

### *In vitro* plant growth promotion assay on bacteria

To assess IAA production, we used the method described by Glickmann and Dessaux (1995), with some modifications for *in vitro* IAA production: we used King B broth with and without additional L-tryptophan (3 g/l), as described by Shokr and Emtiazi (2010). After 6 days of incubation of strain DCY84^T^, IAA production was measured using the colorimetric method (Salkowski reagent). We also prepared several concentrations of IAA to establish the standard curve.

We used *Pseudomonas* Agar F medium (Glickmann and Dessaux, 1995) supplemented with a chrome azurol S (CAS) complex [CAS/iron(III)/hexadecyltrimethylammonium bromide (HDTMA)] to assess siderophore production capacity, as described previously (Schwyn and Neilands, 1987). These three ingredients were prepared separately: we dissolved 60.5 mg CAS in 50 ml distilled water (DW), 72.9 mg HDTMA in 40 ml DW, and 0.0027 g of FeCl_3_-6 H_2_O in 10 ml of 10 mM HCl. After the medium was prepared (in 900 ml DW), we mixed all three ingredients to reach 1 L of volume. Siderophore production can be confirmed by the development of a yellowish to reddish halo zone around colonies in the blue-green medium.

Qualitative testing of the phosphate-solubilizing ability of isolated strains was checked by plate screening methods using the media formulated by (Pikovskaya, 1948). A clear halo region around colonies in the opaque Pikovskaya medium indicated positive results for phosphate solubilization.

### Compatibility of strain DCY84^T^ with *P. ginseng* meyer

Surface-disinfected 2-year-old ginseng roots were used for ginseng pot assay; the roots were dipped in DCY84^T^ suspensions of various ODs (indicating variation in CFUs/ml) for 10 min. Then they were cultivated in sterilized artificial soil (vermiculite:perlite:peat moss = 3:1:1, with additional sterilized tap water [25% (*v/v*)] in pots (11 cm high and 11 cm diameter). We observed rot symptom was development 7 days after post- inoculation and recorded brown rot increased with inoculum population. Each pot contained 7 roots. Each treatment was replicated in three pots.

We performed ginseng disc assay following a protocol with some modifications. 4-year-old of *P. ginseng* roots were disinfected with 70% EtOH for 5 min, 2% NaOCl for 5 min, and then were rinsed twice with sterilized distilled water (SDW). The middle part of the roots was cut transversely to a thickness of 1 cm and a diameter of 2 cm. We cut the bottom and top parts of the roots longitudinally. DCY84^T^ suspensions with several ODs were prepared following the previously described protocol, and 20 μL was given to the cortex of the transverse root sections and the epidermis of the longitudinal root sections. Then we placed the root discs into Petri dishes with sufficient moisture supplied via soaked sterile filter paper and incubated them at 25°C and 30°C. For comparison, we used *P. polymyxa* 10485^T^ as the inoculant because it can cause root rot in ginseng (Kim, 2015). We observed symptom development daily for 7 days after inoculation and then created a symptom severity scale. Each treatment was replicated three times.

### *In vitro* antifungal test against *ilyonectria robusta*

All PGPB candidate strains were activated from stock culture vials on Trypticase soy agar (TSA) and incubated for 2 days at an optimum temperature (25°C or 30°C). All pathogenic fungi were cultured on potato dextrose agar (PDA) medium and incubated at 25°C for 5 days. The following PGPB strains were used in this test: DCY84^T^, *Chryseobacterium yeoncheonense* DCY67^T^, *Epilithonimonas ginsengisoli* DCY78^T^, *Burkholderia ginsengiterrae* DCY85^T^, *B. panaciterrae* DCY85-1^T^, *Sphingomonas panaciterrae* DCY91^T^, *Chryseobacterium* N-15 DCY98^T^, and *S. panacis* DCY99^T^. We also used the following pathogenic fungi: *Ilyonectria robusta* S2-3 and *I. robusta* Hb2 (both isolated from root rot of *P. ginseng*), and *Fusarium solani* KACC 44891^T^. First, all bacteria and fungi were tested for growth on dual media (Supplementary Table S3). The dual media candidates were Waksman agar (WA; peptone 5 g/l, glucose 10 g/l, beef extract 3 g/l, NaCl 5 g/l, agar 20 g/l, pH 6.8), yeast malt agar (peptone 7 g/l, yeast extract 3 g/l, malt extract 3 g/l, glucose 10 g/L, agar 18 g/l, pH 6.2) and tryptone yeast agar (tryptone 5 g/l, yeast extract 3 g/l, calcium chloride 0.66 g/l, agar 18 g/l, pH 7.0). We observed the growth of bacterial colonies and fungi mycelia for 3 and 10 days, respectively. The best medium for both bacteria and fungi was selected for the antifungal test. Fungi isolates were prepared 3 days earlier than bacteria isolates. Plates were divided into 4 parts horizontally using 3 lines, and we streaked bacteria isolates on one quarter line but not the other. After 24 h, a pathogenic fungi mycelia plug (*I. robusta* S2-3, *I. robusta* Hb2, *F. solani* KACC 44891^T^) was placed in the middle line. We used a cork borer to obtain the fungi inoculant plug, which was taken from the edge of fungal colony. The control included only the fungi plug in the middle line without any bacteria streaking. We measured the suppression rate (SR) when the fungi mycelia reached the free-bacteria quarter lines on both the bacteria-streaked and control plates (Farh et al., 2015).

$$\begin{array}{l} \mathrm{SR}=\left[\left(A-B\right)/A\right]\times 100 \end{array}$$

where A indicates the radius of the fungal mycelia away from the bacterial streak (reaching the non-streaked line) and B indicates the radius of the fungal mycelia toward the bacterial streak (reaching the streaked line).

### Optical density and colony forming unit (CFU) correlation of DCY84^T^

DCY84^T^ was grown in Trypticase soy broth at 30°C overnight. The cell culture was centrifuged at 3,000 g for 15 min, and the precipitated cells were dissolved in SDW to a certain OD value, as read by a spectrophotometer at a 600-nm wavelength. We counted CFUs of DCY84^T^ in triplicate via serial dilution of cell suspensions with different OD values. CFU/ml was determined from plate count results after 30°C incubation for 2 days. Correlations between OD and CFU values are shown using linear regression.

### Design of a specific genetic marker for strain DCY84^T^

rRNA was the target of selection when we designed a specific genetic marker for DCY84^T^. From ORFs found and annotated during full genome sequencing (Sukweenadhi et al., 2014), we extracted the 16S rRNA, 23S rRNA, 30S rRNA, and 50S rRNA sequences. For the first screening, we collected up to >500, 500, 400, 300, and 200 bp of nucleotides in front of each ORF sequence and tested their possibilities as promoters using the Promoter 2.0 prediction software (Rozen and Skaletsky, 2000). For each input sequence, we show the name and length first, followed by the Position Score Likelihood in table form, where “position” indicates position in the sequence, “score” indicates the prediction score that a transcription start site occurs within 100 bp upstream from that position, and “likelihood” indicates the descriptive label associated with that score. The score itself always takes the form of positive numbers as follows: <0.5 means ignored; 0.5–0.8 means marginal prediction; 0.8–1.0 means medium likely prediction, and >1.0 means highly likely prediction. We tested the best candidate promoter region from 16S rRNA, 23S rRNA, 30S rRNA, and 50S rRNA again up to >500 bp in front of each ORF sequence and recorded scores above 0.5 (Supplementary Table 7). Considering the size (<1,000 bp) and promoter prediction score, we selected two of the four promoter candidates and designed primers. After optimization of the annealing temperature range from 55°C to 66°C, the best polymerase chain reaction (PCR) conditions were as follows: initial denaturation at 95°C for 2 min; 35 cycles of 95°C for 30 s, 64°C for 50 s, and 72°C for 1 min; then final elongation at 72°C for 5 min. We carried out PCR using 100 ng of DCY84^T^ genomic DNA in a 15-μl total reaction volume of Genotech® 2 × Green PreMix (Genotech, Korea). The PCR mix condition included 1 μl DNA template (100 ng/μl), 1 μl for each primer pair, 7.5 μl Green PreMix, and 4.5 μl of DW for a 15-μl total reaction mix. We verified the species-specific primer for the strain DCY84 compared to other strains such as *P. barengoltzii* KACC 15270^T^, *P. timonensis* KACC 11491^T^, *P. phoenicis* NBRC 106274^T^, *S. asaccharolytica* NBRC 15499^T^, *S. panaciterrae* DCY91^T^, and *B. ginsengiterrae* DCY85-1^T^. As a positive control, we also conducted *nifH* gene PCR.

### Quantification of DCY84^T^ using the specific genetic marker

We used cell suspensions with different OD values as templates (1 μl) in a 15-μl total reaction volume of SYBR® Green Sensimix Plus Master Mix (Watford, England) and performed quantitative real-time PCR (qRT-PCR). Amplification, detection, and data analysis were carried out with a CFX 96/Connect Real-Time PCR system (BIO-RAD, South Korea). We used the following thermal cycler conditions: 2 min at 95°C followed by 40 cycles of 95°C for 30 s, 64°C for 50 s, and 72°C for 1 min. The threshold cycle (Ct) was recorded, and the correlation of OD and Ct values is shown as an exponential regression.

### Assessment of salinity tolerance in *P. ginseng*

Different concentrations of NaCl were prepared, and soil bed was created using water containing NaCl. Plants were exposed to salinity stress in two different periods: at the start of ginseng root cultivation and after the ginseng roots had sprouted (5 days after cultivation, young seedling stage). The treatment was conducted for a short period (3 days) and a long period (12 days) after sprouting, and the roots were harvested for their morphological appearance. Furthermore, the soil was dried in a 50°C oven for 5 days before soil element analysis. For soil element analysis, soil samples were air dried and passed through a 2-mm sieve. We analyzed the chemical properties of the samples according to the standard methods of the Rural Development Administration of Korea (Lee et al., 2016). Soil pH and electrical conductivity (EC) were measured using a soil-to-water ratio of 1:5 with a pH meter (Thermo, Orion 900A) and an Orion 162A conductivity meter, respectively. Soil organic matter content, phosphorus content, and levels of available cations (K^+^, Ca^2+^, Mg^2+^, and Na^+^) were assessed using the Tyurin method, the Lancaster method, and the 1N-NH_4_OAc (pH 7.0) method, respectively.

### RNA extraction and qRT-PCR analysis

The specific primers and annealing temperatures used in qRT-PCR are listed in Supplementary Table 1. At the last step of each cycle, we detected the fluorescent product. The number of cycles at which the fluorescence intensity was significantly higher than background fluorescence during the initial exponential phase of PCR amplification is designated as the Ct. We used the Ct value for the housekeeping gene to normalize the Ct value for the target gene using the formula 2^−ΔΔ*Ct*^ to determine the relative fold differences in template abundance for each sample. The primer efficiencies were determined using the method of Livak and Schmittgen, 2001 to validate the ΔΔCt method. The efficiencies of the gene and the internal control (housekeeping gene) were equal when the observed slopes were near zero. We performed three independent experiments.

### Gas chromatography time-of-flight mass spectrometry (GC-TOF-MS) analysis

We attached an Agilent 7890 GC system (Agilent Technologies, Palo Alto, CA, USA) equipped with an Agilent 7693 auto-sampler to a TOF Pegasus III mass spectrometer (Leco, St. Joseph, MI, USA), which we operated in electron ionization mode (70 eV). The column was an Rtx-SMS column (30 m length × 0.25 mm i.d. × 0.25 μm film thickness; Restek Corp., Bellefonte, PA, USA). The selected carrier gas was helium, which we maintained at a constant flow of 1.5 mL/min. Then we injected 1 μL of the derivative sample in a split mode (10:1). The oven temperature was maintained at 75°C for 2 min, increased to 300°C at a rate of 15°C/min, and then held at 300°C for 3 min. We set the acquisition rate to 20 scans/s with a mass scan range of 45–1,000 *m/z*. The injector and ion source temperatures were set at 250°C and 230°C, respectively (Lee et al., 2016).

GC-TOF-MS raw data were converted to netCDF format (^*^.cdf) using ChromaTOF software (LECO). After conversion, the MS data were processed using Metalign software (Lommen, 2009), and the resulting data were exported to an Excel (Microsoft, Redmond, WA, USA) file. Multivariate statistical analyses were processed using SIMCA-P+ (version 12.0, Umetrics, Umea, Sweden). We performed principal component analysis (PCA) and partial least-square discriminant analysis (PLS-DA) to compare metabolite differences between samples. Orthogonal projection to latent structures-discriminant analysis was also performed to compare metabolite differences between the aerial part (circle, O) and the root (triangle, Δ). Significantly different metabolites between the two samples were selected using Variable Importance in Projection (VIP) values > 0.7 and *p* < 0.05 as cutoffs.

### Chlorophyll and carotenoid content in leaves

We determined chlorophyll and carotenoid content spectrophotometrically according to a previously described method (Lichtenthaler, 1987). First, 300 mg of leaves were ground into a powder with liquid nitrogen and then transferred to a 15-ml Falcon tube. We added 5 mL of 80% acetone to the tube, mixed thoroughly, and stood it in the dark overnight. Centrifugation was performed at 4°C for 15 min (2,500 rpm). We transferred the supernatant to a new centrifuge tube and measured the absorbance of chlorophyll (hereafter termed A) using spectrophotometry. The chlorophyll and carotenoid concentrations were calculated as follows (using 80% acetone as a blank control): Chlorophyll a = 12.72^*^A_663_-2.59^*^A_645_; Chlorophyll b = 22.9^*^A_645_-4.67^*^A_663_; Total chlorophyll = 20.31^*^ A_645_+8.05^*^ A_663_; Carotenoid = A_480_ + (0.114^*^A_663_-0.638^*^A_645_).

### Hydrogen peroxide (H_2_O_2_) and superoxide (${\text{O}}_{2}^{-}$) visualization

We used 3,3′-diaminobenzidine (DAB) staining for *in situ* detection of H_2_O_2_. The harvested leaf samples were placed in a Petri dish. Three milliliters of DAB staining solution (50 mg DAB in 45 ml DW with the addition of 25 μl of Tween 20 and 2.5 ml of 0.2M Na_2_HPO_4_) was applied to the leaves until they were fully immersed. As the control treatment, we applied 2 ml of 10 mM Na_2_HPO_4_ to leaves. The Petri dishes were placed in a desiccator and gently vacuumed for 5 min. Because DAB is light-sensitive, we covered the Petri dish with aluminum foil. The Petri dish was shaken on a standard laboratory shaker for 3 h at 100 rpm. We removed the foil after the incubation and used a bleaching solution (ethanol: glycerol: acetic acid = 3:1:1) to replace the DAB staining solution. After boiling for 15 min, we added fresh bleach solution to replace the used bleach solution. After an additional 30 min at room temperature, we took photographs under uniform lighting.

*In situ* accumulation of superoxide anions (${\text{O}}_{2}^{-}$) was examined by histochemical staining with nitro blue tetrazolium (NBT). We placed the samples in the NBT solution (0.1 mM NBT, 25 mM HEPES pH 7.6) and subjected them to vacuum infiltration for 5 min. After incubation in the dark for 2 h, we treated the samples with 80% ethanol and then took photographs under uniform lighting.

### Soil enzyme activity assay

We measured soil enzyme activity in fresh, moist soil. Dehydrogenase activity was measured following Pepper et al. (1995) through the reduction of 2,3,3 triphenyltetrazolium chloride and expressed as μg triphenylformazan/g soil. Alkaline phosphatase activity was estimated using the method of Eivazi and Tabatabai (1977), whereas amylase and invertase activity was measured following the method developed by Cole (1977).

### Relative water content (RWC) measurement

We measured RWC on the day of sampling. We recorded the fresh weight (FW) of both shoots and roots using the following formula:

$$\begin{array}{l} RWC=(fresh\;weight-dry\;weight)/(turgid\;weight\\ \;-dry\;weight)\times 100\% \end{array}$$

The turgid weight was determined by keeping the shoot and root in distilled water in darkness at 4°C until they reached a constant weight (overnight). Dry weight was obtained 72 h after keeping the turgid leaf in an oven at 70°C.

### Measurement of proline content

Proline was determined as previously described with slight modifications (Woodrow et al., 2017). Proline extraction was performed using 50x diluted fresh weight (*w/v*) in a mixture of 70:30 ethanol:water (*v/v*). Extracts were mixed with an equal volume of reaction mix (ninhydrin 1% (*w/v*) in ethanol:acetic acid:water = 20:60:20 (*v/v*)) under light-protected conditions. The reaction mixture was placed in a 95°C water bath for 20 min. After cooling to room temperature, it was spun down quickly for 1 min at 2,500 rpm. We then read the absorbance at 520 nm and calculated the amount of proline in the extracts as follows:

$$\begin{array}{l} Proline(\mu g/mol/gFW)=\frac{\left(Abs_{extract}-blank\right)}{\;}\times \frac{Vol_{extract\_}}{Slope}\\ \;\times \frac{1}{Vol_{aliquot}FW} \end{array}$$

Abs_extract_ is the extract absorbance value; blank (expressed as absorbance) and slope (expressed as absorbance/nmol) are specified through linear regression; Vol_extract_ is the extract total volume; Vol_aliquot_ is the volume used in the assay; and FW (expressed in mg) is the amount of plant material used in the extraction process. We presume that Abs_extract_ is within the linear range. As standards, we prepared proline solutions ranging from 0.04 to 1 mM in the same medium used for the extraction.

### Total soluble sugar content analysis

The TSS content of the dried plant material was determined as described previously (Irigoyen et al., 1992).

### Quantitative MDA and H_2_O_2_ measurement

Lipid peroxidation (LPO) was measured using thiobarbituric acid (TBA) assay according to the method described by Buege and Aust (1978). Lipid peroxides were extracted by grinding 500 mg of leaves with an ice-cold mortar and 6 ml of 100 mM potassium phosphate buffer (pH 7). Homogenates were filtered through one Miracloth layer and centrifuged at 15,000 g for 20 min. Chromogen was formed by mixing 200 μl of supernatant with 1 ml of a reaction mixture containing 15% (w/v) trichloroacetic acid (TCA), 0.375% (w/v) 2-thiobarbituric acid (TBA), 0.1% (w/v) butylhydroxytoluene, and 0.25 N HCl and incubating the mixture at 100°C for 30 min. After cooling at room temperature, tubes were centrifuged at 800 g for 5 min, and the supernatant was used for spectrophotometric readings at 532 nm. We estimated lipid peroxidation as the content of 2-thiobarbituric acid-reactive substances (TBARS) and expressed it in equivalents of malondialdehyde (MDA). The calibration curve was made using MDA in the range of 0.1–10 nmol. A blank for all samples was prepared by replacing the sample with extraction medium, and controls for each sample were prepared by replacing TBA with 0.25 N HCl. In all cases, 0.1% (*w/v*) butylhydroxytoluene was included in the reaction mixtures to prevent artifactual formation of TBARS during the acid-heating step of the assay (Rael et al., 2004).

Fresh leaf tissues (100 mg) were homogenized with 1 ml 0.1% (*w/v*) TCA and then centrifuged at 12,000 g for 15 min. An aliquot of the supernatant (250 μl) was added to 250 μl of 10 mM phosphate buffer (pH 7.0) and 500 μl of 1 M potassium iodide (KI) to make 1 ml total volume. The absorbance of this mixture at 390 nm and calculated the H_2_O_2_ content using a standard curve with concentrations ranging from 0.05 to 0.1 mM (Junglee et al., 2014).

### Crude protein extraction and ROS scavenging enzyme activity assay

Fresh leaf tissue (0.2 g) was homogenized with 2 ml of 50 mM cold phosphate buffer (pH 7.8) and centrifuged at 12,000 × *g* for 20 min at 4°C. We collected the supernatant for further determination of the activities of ROS scavenging enzymes. The peroxidase activity was measured by an oxidation reaction between H_2_O_2_ and guaiacol; catalase (CAT) activity was measured based on the d409-421)ecomposition of the H_2_O_2_ content, and ascorbate peroxidase (APX) activity was measured based on the reduction of ascorbate. We used an ELISA reader (Synergy-2, Bio-Tek Instruments, Inc., Winooski, VT, USA) to read the absorbance at specific wavelengths and a Coomassie (Bradford) protein assay kit to determine the protein concentrations in the samples. One unit of enzyme activity was defined as the amount of enzyme that oxidized 1 μM of substrate per min at 25°C (μl/mg protein/min). We determined enzyme activity by measuring the change in absorbance per minute, divided by the amount of protein (mg) as follows:

(1) $$Enzyme\;activity=\frac{\left(A2-A1\right)}{\left(T2-T1\right)}\times \frac{1}{mgprotein}$$

### Statistical analysis

All experiments were performed using three biological triplicates. Statistical analyses were carried out using GraphPad Prism 5 software program (GraphPad Software, La Jolla, CA). The data from three biological groups were represented as mean ± SE and analyzed using Student *t*-test. All significant values are presented as ^*^p < 0.05, ^**^p < 0.01, and ^***^p < 0.001 respectively.

## Results

### Screening and properties of plant growth-promoting traits isolated from ginseng soil

We checked the *in vitro* plant growth-promoting properties of selected strain candidates and their closest reference strains and summarized them in Supplementary Table 2. The newly re-classified genus *Paenibacillus* was rarely studied for plant growth-promoting activity, except for *P. polymyxa*, which has been commercialized. DCY84^T^ was found to produce 52.9 ± 1.85 IAA (without L-tryptophan) and 72.83 ± 2.86 μg/ml IAA (with L-tryptophan). Apart from this, DCY84^T^ also produced siderophore and was positive on the phosphate solubilization test. The other tested strains were not as effective as DCY84^T^ (Supplementary Table 2). Our results suggest that DCY84^T^ has no antifungal activity (negative SR) against our selected pathogenic fungi (Supplementary Figure 1). In our preliminary check, all strains of bacteria and fungi were tested on different media. The best medium for supporting the growth of bacteria and fungi was the WA medium, as summarized in Supplementary Table 4. We selected *P. yonginensis* DCY84^T^ for further study because of its strong *in vitro* plant growth-promoting activity.

### Quantification of strain DCY84^T^

To prepare the bacterial inoculant, we made some needed adjustments to simplify the process. By finding correlations between OD (read at a 600-nm wavelength) and CFUs/ml, we were able to prepare the desired amount of bacterial inoculant without waiting to count colonies after plate streaking. The equation for DCY84^T^ is y = 4.6396x + 2.0996, with *R*^2^ = 0.9926, and the equation for KACC 10485^T^ is y = 8.7844x + 0.8931, with *R*^2^ = 0.9944, where x indicates the OD value at 600 nm, and y indicates the log (CFU/ml). The relationship between OD and CFU is shown in Supplementary Table 4.

Furthermore, to estimate bacterial distribution after DNA sampling from either bulk soil or rhizospheric soil, we developed a specific marker using the DCY84^T^ genome. Thus, we could quantify the number of bacteria from the Ct value on real-time PCR. From the full DCY84^T^ genome (Kim et al., 2017), we annotated 8 sequences of 16S rRNA, 3 sequences of 23S rRNA, 22 sequences of 30S rRNA, and 34 sequences of 50S rRNA. After screening each of the promoter regions, we selected the highest promoter prediction value for each rRNA for further analysis. In the end, we selected the two best promoter candidates based on their prediction values and allowable size: p16S rRNA-5′ (TTC CGA AGG ATA TAT CGG) and p16S rRNA-3′ (CGG CAC TTA CCC C) derived from the 694-bp promoter region of orf05875 and annotated as 16S rRNA methyltransferase and p30S rRNA-5′ (TCT TTG CAG CAG CCC CTC TAT) and p30S rRNA-3′ (ACC GTA GTT GCT GAT C) derived from the 735-bp promoter region of orf03322 and annotated as 30S ribosomal protein s4. After annealing optimization, we tested the respective primers, and the results are shown in Supplementary Figure 2A. The 16S rRNA DCY84^T^ promoter primers were highly specific to DCY84^T^. On the other hand, the 30S rRNA DCY84^T^ promoter primers can produce the right amplicon from the DCY84^T^ sample, but they are not specific; many amplicons were generated from other bacterial DNA samples. The full genome sequences of strain DCY84^T^ was deposited in NCBI (PRJNA306396).

We used qRT-PCR with the selected 16S rRNA DCY84^T^ promoter primers for further specificity checking by looking at its melt curve and melt peak, as visualized in Supplementary Figure 2B. Neither the curve nor the peak was detected in other bacterial samples or the water used as the template. Using these primers, we made a standard curve from the qRT-PCR results by using various DCY84^T^ suspensions (different OD values). Thus, in the end, we were able to correlate the Ct value with the CFU/mL of DCY84^T^. We generated an equation for DCY84^T^ OD values (x) and Ct values (y): y = −0.7068x + 23.581, *R*^2^ = 0.993. The conversion of OD values into Ct values and predicted CFUs/mL are shown in Supplementary Table 5.

### Compatibility of strain DCY84^T^ with *P. ginseng* meyer

**Figure 1:**
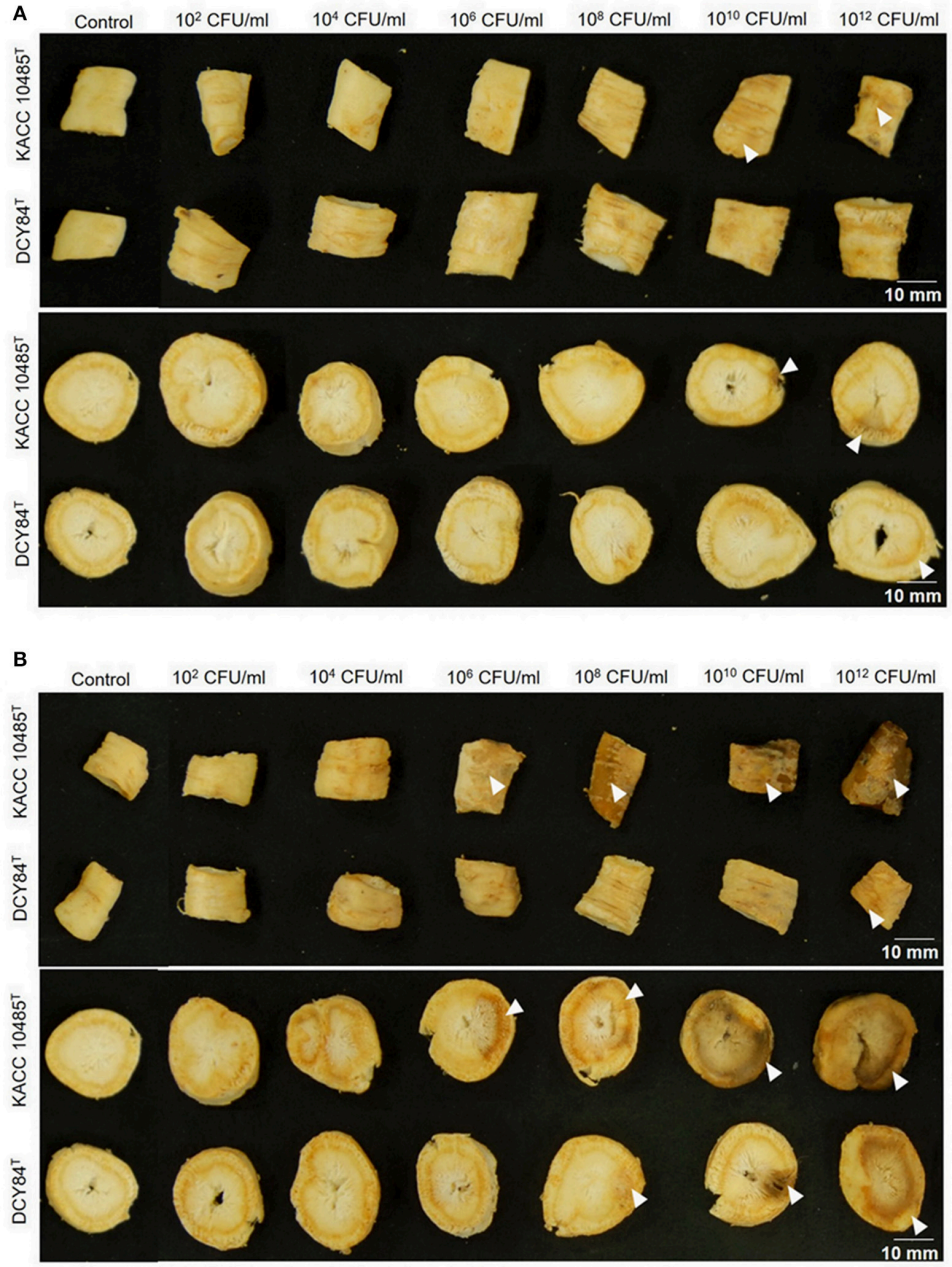
Ginseng disc assay for *in vitro* compatibility testing of strain DCY84^T^ on *P. ginseng*. **(A,B)** Symptoms in both the cortex and epidermis were observed 7 days after bacterial treatment using various CFU/mL inoculants and incubated at 15°C **(A)** and 30°C **(B)**. *P. polymyxa* KACC 10485^T^ and *P. yonginensis* DCY84^T^ were used in this test. The white arrow indicates the position of the symptom. White scale bar indicates 10 mm.

**Figure 2:**
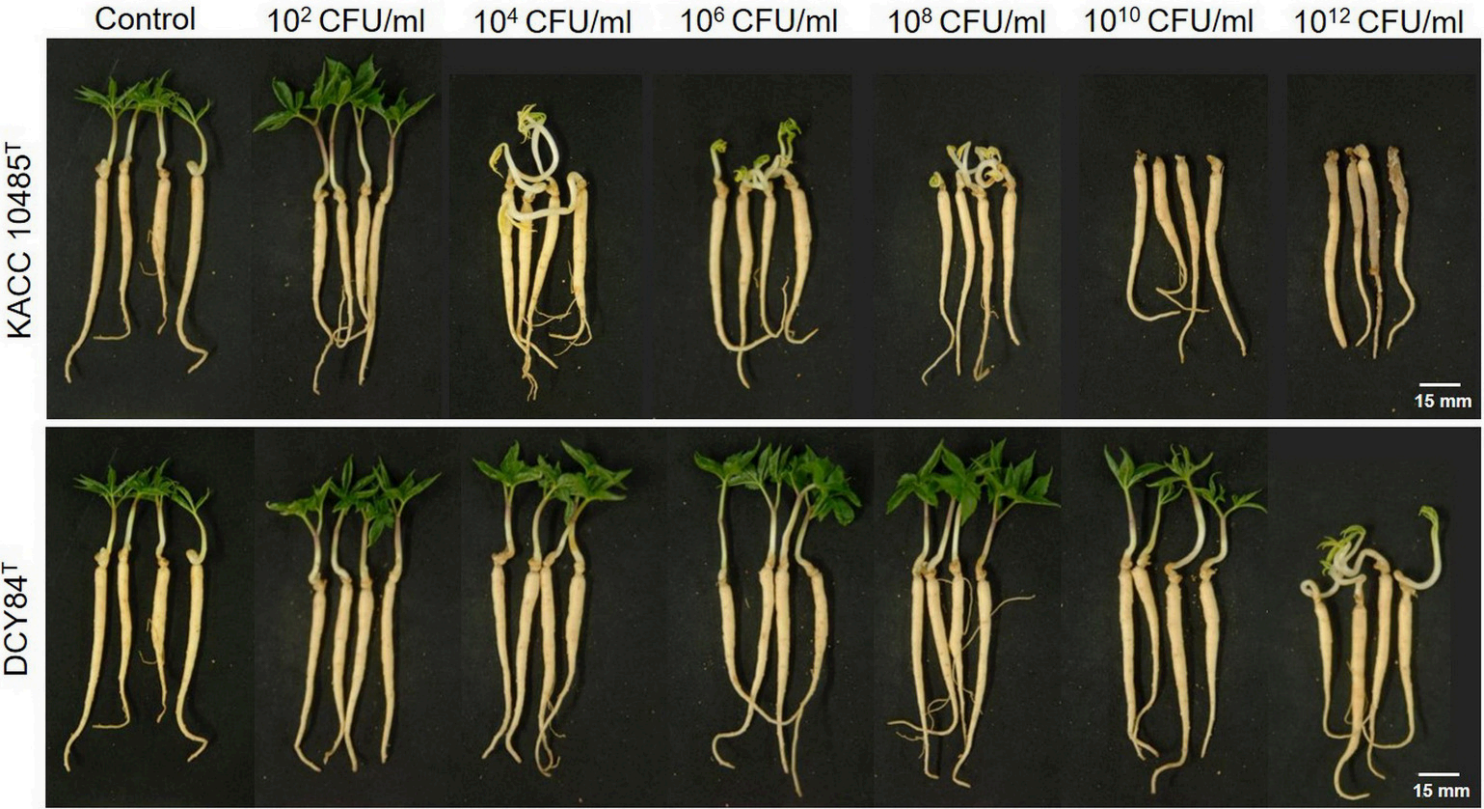
Ginseng pot assay for *in planta* compatibility testing of strain DCY84^T^ on *P. ginseng*. Symptoms in ginseng seedlings were measured 7 days after bacterial treatment using various CFU/mL inoculants in a soil system. *P. polymyxa* KACC 10485^T^ and *P. yonginensis* DCY84^T^ were used in this test. Root rot symptoms did not appear, but some stressed/redundant growth was observed. White scale bar indicates 15 mm.

**Figure 3:**
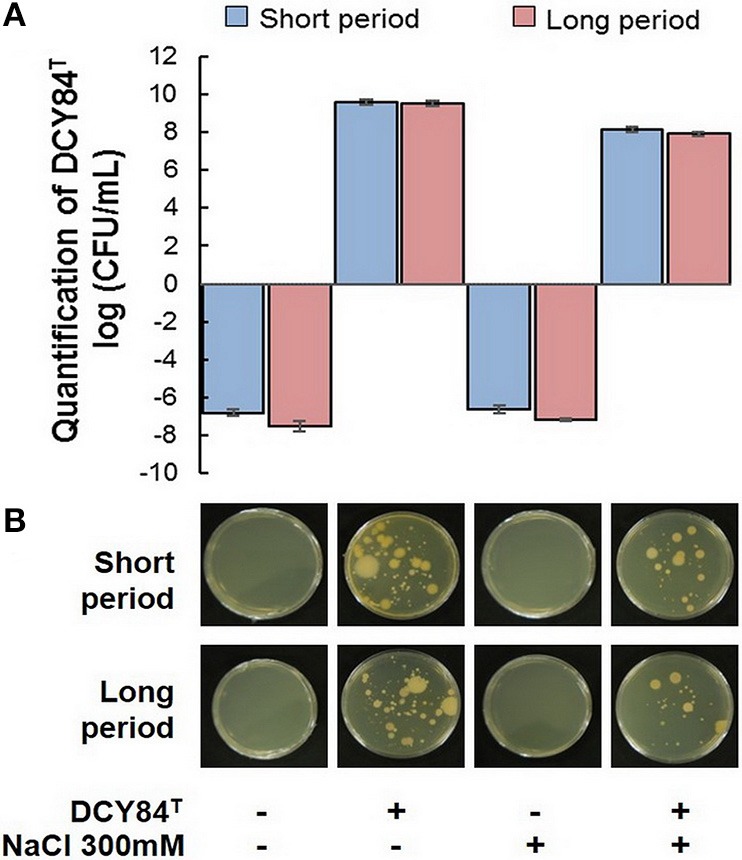
Monitoring of strain DCY84^T^ in the *P. ginseng* rhizosphere. **(A)** Based on specific markers, DCY84^T^ can be detected in a soil DNA sample. Although DCY84^T^ was abundant, some bacterial contaminants were also present, as revealed by **(B)** plate streaking. The small colony with a white yellowish color is strain DCY84^T^ (white arrow). The large colony with a yellow/white color is a bacterial contaminant.

We measured the *in vitro* compatibility of DCY84^T^ and *P. ginseng* using the disc test (Figure 1) and the severity scale (Supplementary Figure S3). After 7 days of bacterial treatment, changes in symptoms were observed and compared with those from *P. polymyxa* KACC 10485^T^ *in vitro* (Figure 2) and *in planta* (Figure 3). The pot assay (*in planta*) yielded distinctly different results from *in vitro* test (the root disc), depicting that the strain DCY84 might interact with more biocompatibility in a real ecosystem. Root rot symptoms did not appear in the pot assay (except for the 10^12^ CFU/mL *P. polymyxa* treatment) as clearly as they did on the *in vitro* test. However, the roots showed some stress symptoms in the sprouting stage.

Although the results were different, they had a similar pattern: the roots showed either rot or stress symptoms when the amount of bacterial inoculant was high. The *in vitro* assay indicated that 10^8^ CFU/mL was the safest DCY84^T^ dosage for ginseng treatment (*P. polymyxa* at 10^6^ CFU/mL). Meanwhile, the *in-planta* assay indicated that DCY84^T^ can be given to ginseng seedlings up to 10^12^ CFU/mL for 10 min (*P. polymyxa* at 10^10^ CFU/mL). Summaries of both tests are shown in Table 1.

**Table 1:** Summary of ginseng compatibility results.

Inoculum concentration^*^	**Ginseng disc assay**		**Ginseng pot assay**	
	**DCY84^T^**	**KACC 10485^T^**	**DCY84^T^**	**KACC 10485^T^**
Control	–	–	–	–
10^2^ CFU/mL	–	–	–	–
10^4^ CFU/mL	–	±	–	–
10^6^ CFU/mL	–	±	–	–
10^8^ CFU/mL	–	+	–	–
10^10^ CFU/mL	±	*++*	–	–
10^12^ CFU/mL	+	*++*	–	±

* Inoculum number calculated using the chart in Supplementary Table 5.

*4-level severity scale: –, no symptoms; ±, yellowish discoloration or small lesion formation; +, mild brown rot, root tissue starting to become soft; and ++, severe brown rot, root tissue soft*.

### Assessment of salinity tolerance in *P. ginseng*

The growth of 2-year-old ginseng seedlings was arrested when NaCl solution was given before the seedlings sprouted. Also, 300–450 mM NaCl solutions affected ginseng seedlings after they sprouted (see Supplementary Figure4). Further soil element analysis (see Supplementary Table 6) showed that adding 300 mM NaCl in water to the soil system was enough to yield >1.5 dS/M of salinity, which is the point at which salinity stress started to occur in the ginseng seedlings.

### Viability of strain DCY84^T^ in the *P. ginseng* rhizosphere after inoculation

**Figure 4:**
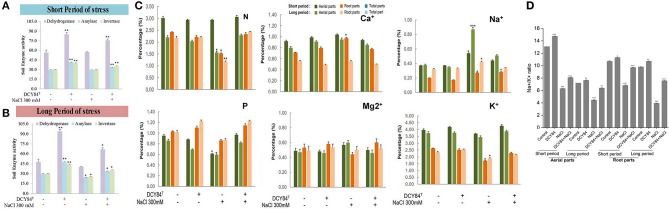
Enzymes, ion and mineral content after short- and long-term salinity stress. Ion, mineral and enzymes content was measured after sample collection. (1) Control; (2) DCY84^T^ treatment; (3) NaCl 300 mM treatment; (4) DCY84^T^ + NaCl 300 mM treatment. **(A,B)** Dehydrogenase activity: Formation of triphenylformazan (g) from TTC/g soil for 24 h. Amylase activity: Glucose (g) liberated from starch/g soil for 48 h. Invertase activity: Glucose (g) liberated from sucrose/g soil for 6 h. Enzymes analysis was conducted using root tissue samples. **(C)** For ion profiles, we used 20 seedlings from each treatment per replication. **(D)** Na^+^/K^+^ ratio was measured in aerial and root parts after treatments. Statistical significance was assigned using the student *t*-test (^*^P < 0.05, ^**^P < 0.01, and ^***^P < 0.001) from the mean of three biological replicates.

First, we assessed DCY84^T^ abundance in the ginseng root rhizosphere using specific PCR markers. DCY84^T^ was quite abundant compared with the starting amount (log CFU/mL = 10, CFU/mL = 10^10^). Despite salinity stress, the number of DCY84^T^ was not significantly reduced. Because no surface sterilization was done before culturing the ginseng seedlings, other bacterial contaminants were found in the PCR product formed by the *fimH* universal primers. The presence of microbial contaminants was also visualized using streaking serial dilution of the same rhizosphere soil sample. The morphological differences in the contaminants made them easy to distinguish from the DCY84^T^ colonies. The contaminants were not abundant (minority), and they decreased following salinity stress (Figure 3). Dehydrogenase, amylase, and invertase activity were higher with DCY84^T^ treatment regardless of salinity stress progression. Moreover, those activities corresponded with DCY84^T^ abundance, indicating that DCY84^T^ enriched soil system nutrients (Figures 4A,B). During short-term of salinity stress, we found a higher content of N and P in both the aerial and root parts of DCY84^T^-treated ginseng after salinity stress treatment than in non-treated stressed ginseng. Under long-term stress conditions, lower N and P content was observed in both the aerial and root parts; when the stressed seedlings were primed with DCY84^T^ treatment, a gradual increase in accumulation of N and P content could be seen (Figure 4C).

### Osmolyte production as defense mechanism against osmotic stress

**Figure 5:**
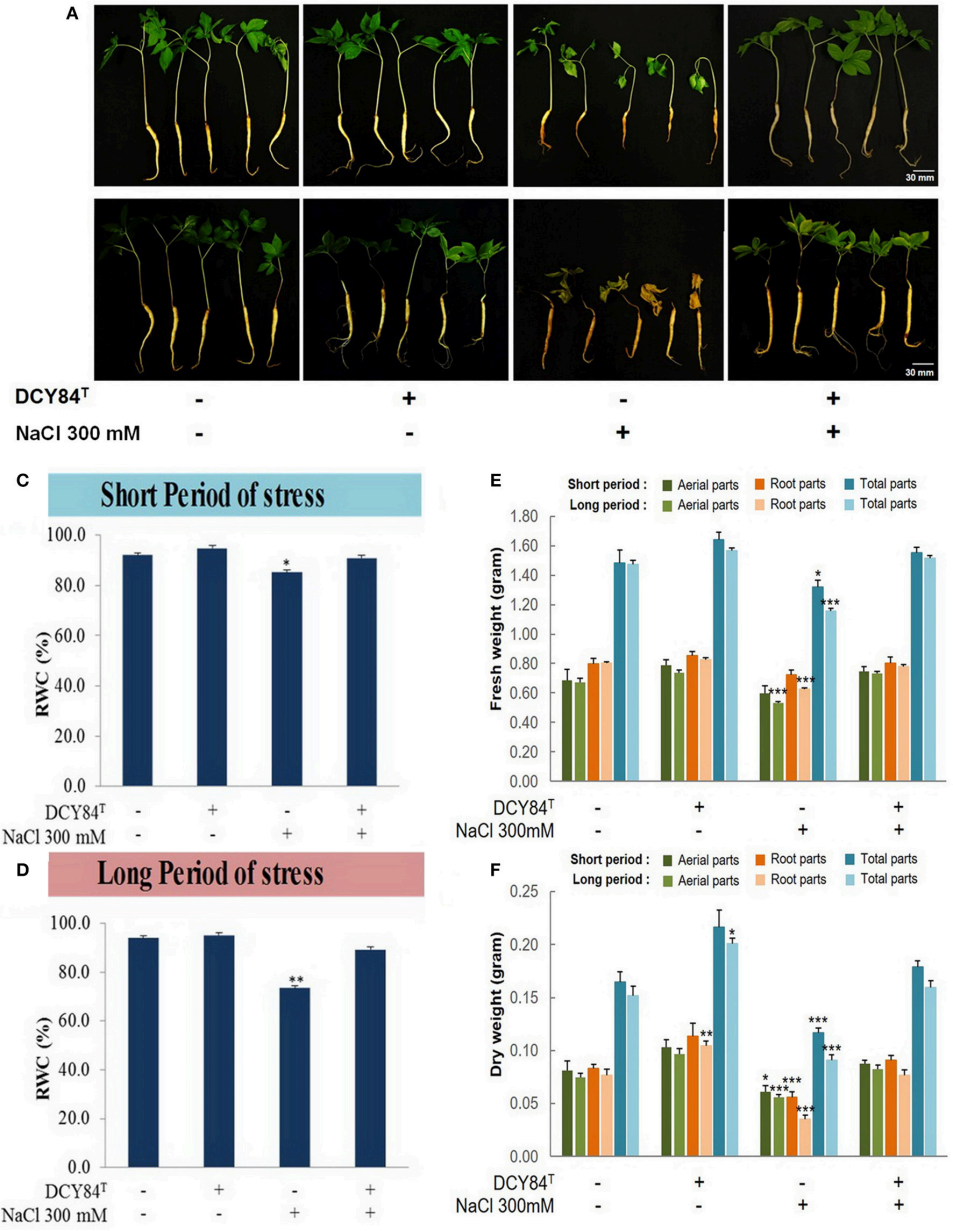
Morphological appearance and biomass profiles of *P. ginseng* after short and long periods of salinity stress. **(A)** After short-term (3 days) salinity stress, the stressed plant started showing root color changes and shrinkage, followed by turgidity loss in the aerial parts. The primed seedlings showed no visible stress symptoms. **(B)** After long-term (12 days) salinity stress, the stressed plants withered almost completely, with dried aerial parts and dead roots. The ginseng seedlings primed with DCY84^T^ grew redundantly with pale green leaves. **(C,D)** Relative water content (RWC) of *P. ginseng* seedlings under short and long-term salinity stress. Salinity stress instantly reduced turgidity, as revealed by lower % of RWC. **(E)** Fresh weight and **(F)** dry weight were measured from aerial parts, root parts and total seedlings. We measured 20 seedlings from each treatment per replication. Data represent the mean ± SE of three biological replicates and statistical significance was assigned using the student *t*-test (^*^P < 0.05, ^**^P < 0.01, and ^***^P < 0.001).

During the short period of salinity stress and without DCY84^T^ inoculation, the aerial part of the stressed ginseng seedlings withered, indicating turgidity loss that caused water/osmotic stress. Meanwhile, some discoloration started happening in the roots (Figures 5A,B). A decrease in the relative water content (%RWC) in the aerial part denotes that significant water loss occurred (Figures 5C,D). However, inoculation of the stressed plants with strain DCY84^T^ resulted in maintenance of the moisture content in the plant tissue, thereby showing enhanced salt tolerance by maintaining RWC to control levels even under stress conditions (Figures 5E,F).

**Figure 6:**
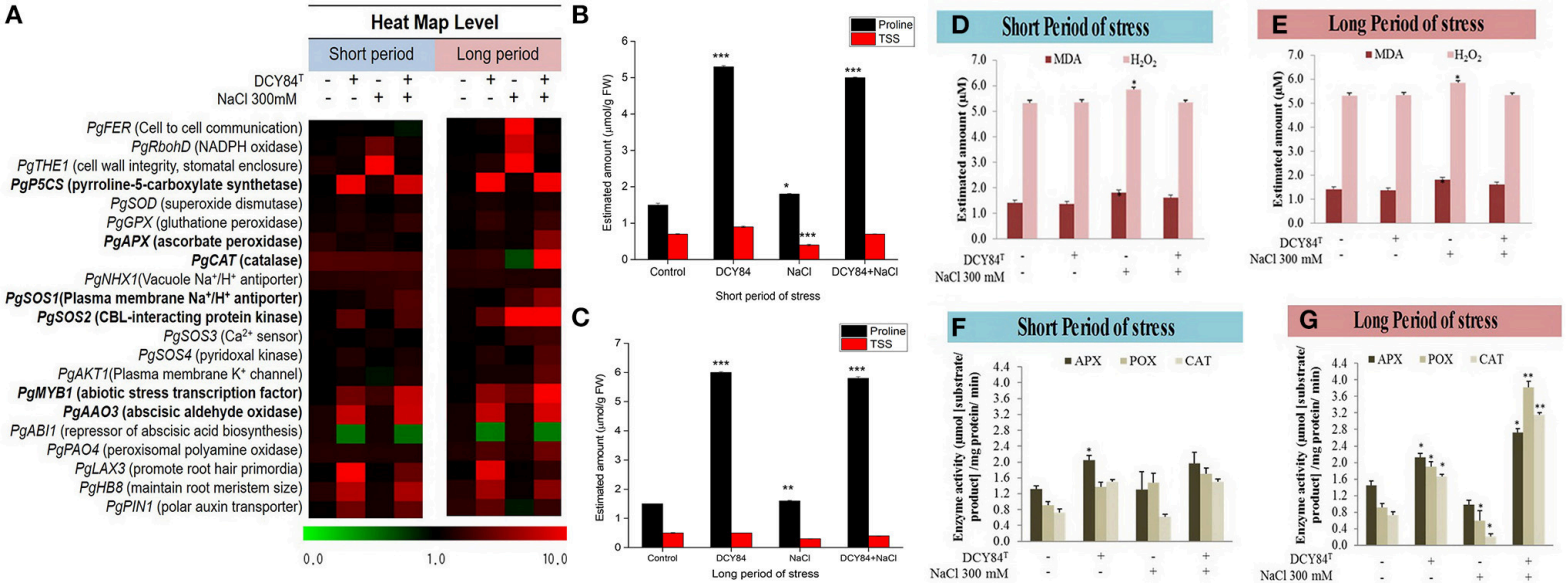
Priming of DCY84^T^ alleviates various biochemical changes caused by short- and long-term salinity stress. **(A)** Heat map of qRT-PCR results for salinity-responsive genes. The relative expression of ginseng genes was normalized using *PgCYP* as the housekeeping gene. Bold letters indicate significant fold change in relative expression from at least one kind of treatment. **(B,C)** Proline and total soluble sugar (TSS) levels of *P. ginseng* seedlings. **(D,E)** MDA and H_2_O_2_ levels of *P. ginseng* seedlings. Both assays were conducted using root tissue samples. **(F,G)** Antioxidant enzyme activity of *P. ginseng* seedlings. Data represent the mean of three biological replicates and were analyzed using the student *t*-test (^*^P < 0.05, ^**^P < 0.01, and ^***^P < 0.001). Short period, 3 days of salinity stress; long period, 12 days of salinity stress.

The ginseng therefore probably increased osmolyte production such as simple sugars, proline, and polyamine. One defense mechanism against osmotic stress in the DCY84^T^-treated seedlings was proline production (Figures 6B,C). We observed the increase in transcription level of *PgP5CS* (Figure 6A), which was consistent with an accumulation of proline (Figures 6B,C) in DCY84^T^-treated seedlings. Total soluble sugar content (TSS) increased significantly in the stressed ginseng, indicating the plants efforts to maintain homeostasis. However, the level of TSS in the DCY84^T^-primed seedlings did not alter significantly when the plants were stressed.

### Ion pump activation on ionic stress

As salinity stress progresses, ionic stress can occur through Na^+^ toxicity. We found that several ion pump genes were highly expressed, including *PgSOS1, PgSOS2, PgSOS3, PgSOS4*, and *PgAKT1* but not *PgNHX1* (Figure 6A). Among them, *PgSOS2* was found to be the most expressed transcript in DCY84^T^-treated seedlings with or without exposure to salinity stress. The ion profile showed a similar pattern of results: the primed ginseng had more K^+^ and less Na^+^ than the stressed plants. We found more Ca^2+^ in the stressed ginseng roots, but no significant change in Mg^2+^. Ca^2+^ presence might directly influence and regulate the osmotolerance of cell membranes under salinity stress. Overall, we found higher ion content in the aerial parts than in the roots (Figure 4C).

### ROS scavenging activity as a defense mechanism against oxidative stress

**Figure 7:**
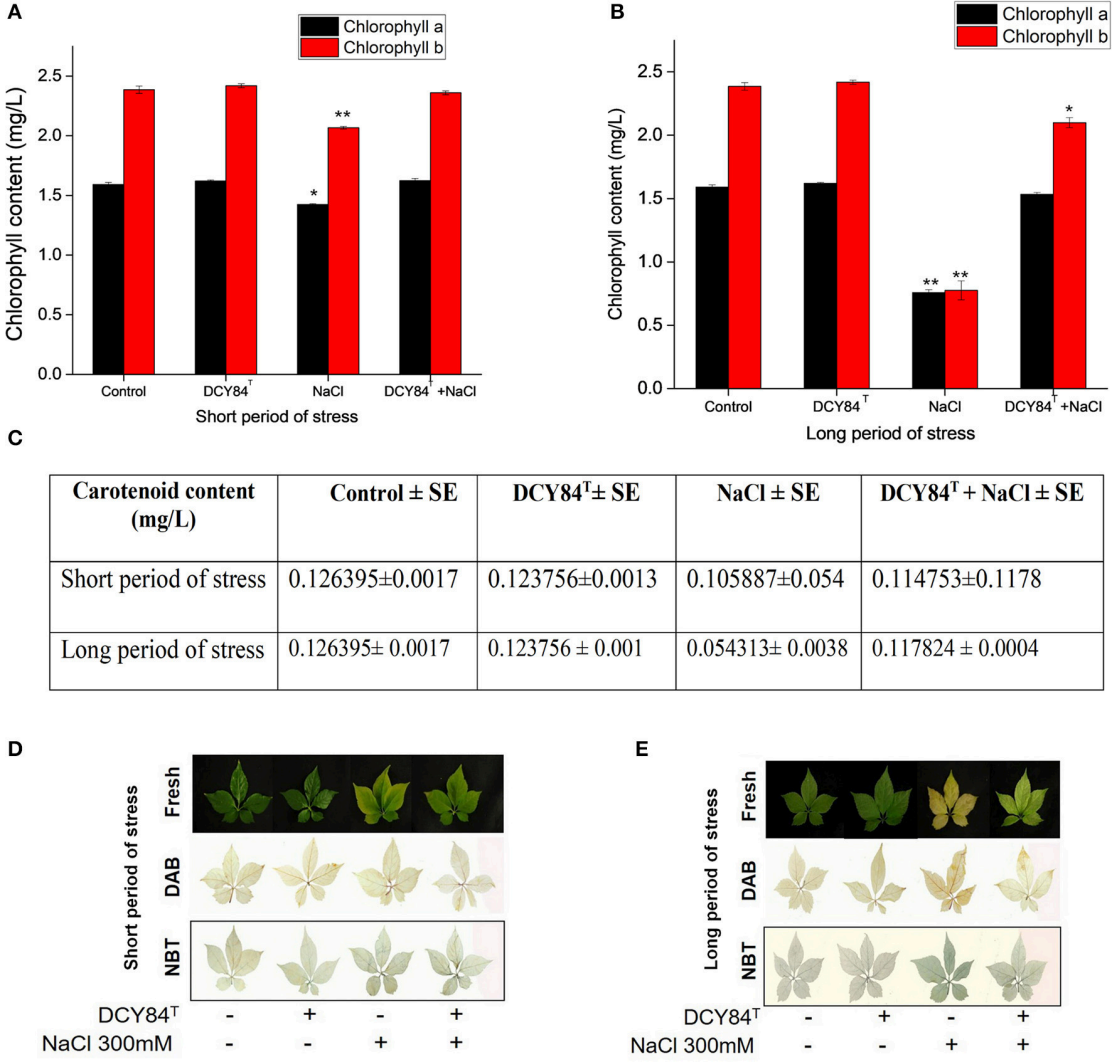
Chlorophyll content, DAB and NBT staining of *P. ginseng* leaves. All assays used fresh samples of ginseng leaves after **(A)** chlorophyll content during short period and **(B)** long period of salinity stress; **(C)** carotenoid content during short and long period of stress; **(D,E)** H_2_O_2_ accumulation showed dark brown color on DAB staining, and superoxide accumulation showed a dark blue color on NBT staining. The statistical significance of three biological replicates was determined using the student *t*-test (^*^P < 0.05 and ^**^P < 0.01).

The increase in the *PgRboHD* transcript might have resulted from oxidase-dependent ROS accumulation (Figure 6A) (Morales et al., 2016). Excessive ROS might have activated cell-to-cell signaling as a warning message to other cells, resulting in the accumulation of the *PgFE* transcript (cell-to-cell communication) (Figure 6A). Furthermore, high H_2_O_2_ (ROS) was found in stressed plants, along with MDA, a lipid oxidation product (LOP) marker (Figures 6D–F). ROS accumulation might have increased the transcription of several antioxidant genes significantly, including *PgAPX* and *PgCAT*, but not *PgSOD* and *PgGPX* (Figure 6A). The transcription level correlated with the activities of antioxidant enzymes in the ginseng seedlings (Figures 6F,G). During short-term salinity stress, the aerial parts of ginseng seedlings exhibited decreased chlorophyll, carotenoids and accumulated ROS in leaves. However, DCY84^T^-primed seedlings were able to recover from salt stress, displaying increased chlorophyll, carotenoid and reduced ROS accumulation compared to non-treated seedlings (Figures 7A,C,D). As the salinity stress progressed, the aerial part of the stressed ginseng dried and withered. However, the primed seedlings showed only pale green leaves, as seen in Figure 7E. This phenomenon was due to either oxidative or ionic stress, which occurs during long-term salinity stress. Therefore, we found a significant decrease in chlorophyll and carotenoid levels in stressed ginseng during the long period of salinity stress (Figure 7B). Meanwhile, in the primed seedlings, the leaves started to become a pale green color (Figure 7E). This symptom corresponded with a slight decrease in chlorophyll during long-term salinity stress (Figure 7B). The reduction in chlorophyll and ROS accumulation in leaves were observed on DAB and NBT staining of ginseng leaves (Figure 7E). The symptoms became more severe as salinity stress progressed. On the other hand, ginseng seedlings primed with DCY84^T^ showed only a slight decrease in chlorophyll content and ROS accumulation compared to stressed plants (Figures 7A–E).

### Metabolite profile of *P. ginseng*

**Figure 8:**
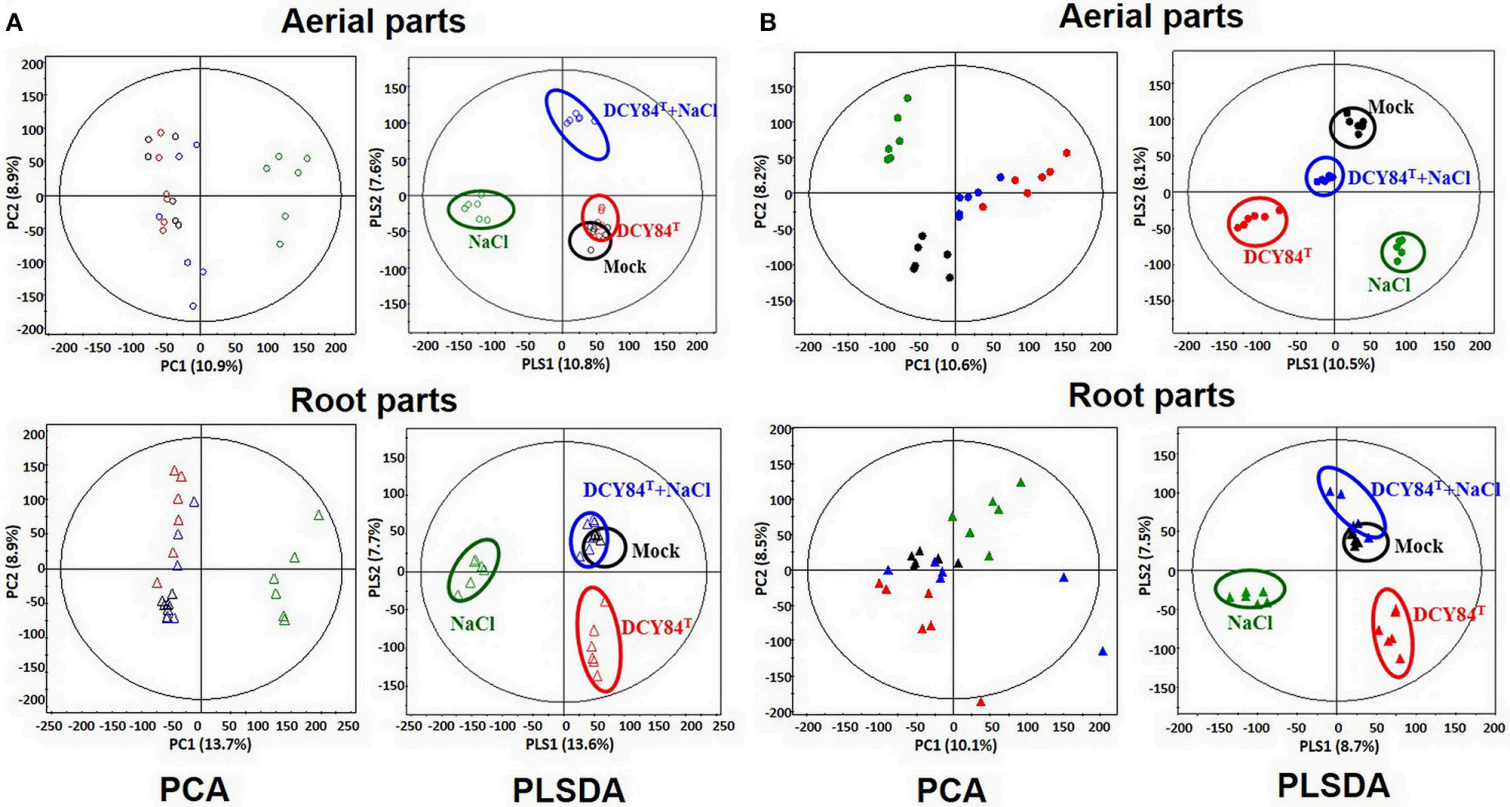
PCA and PLS-DA score plots from *P. ginseng* samples. We analyzed the aerial and root parts of *P. ginseng* separately after **(A)** short period and **(B)** long period of stress exposure. By using gas chromatography time-of-flight mass spectrometry (GC-TOF-MS), the PCA and PLS-DA analysis of metabolites are presented. O, aerial part; Δ, root part; Black icon, Mock; Red icon, DCY84^T^ treatment; Green icon, NaCl 300 mM treatment; Blue icon, DCY84^T^ + NaCl 300 mM treatment.

The results of our GC analysis showed differences in the aerial parts of ginseng plants over time. Based on PCA and PLS-DA analyses, the metabolites produced in the Mock and DCY84^T^ treatments were similar during short-term stress and unlike those from the other treatment conditions (Figure 8A). Furthermore, all metabolites differed from one another as salinity stress progressed (Figure 8B). On GC-TOF-MS analysis, PC1 showed distinct differences between shoots and roots. PC2 could be used to differentiate short-term (3 days) and long-term (12 days) salinity stress treatment. Short-term roots/long-term roots/long-term roots/long-term buds could be identified on PLSDA. PLSDA presented the metabolites showing significant differences in VIP > 0.7 and *p*-values > 0.05 for each group. Primed ginseng roots under salinity stress produced metabolites closer to those under mock treatment than to those from the stressed group.

## Discussion

Salinity is the most severe environmental cause of abiotic stress and disrupts crop production in all cultivated lands. *Panax ginseng* is a medicinal plant requiring long-term cultivation that is prone to various environmental factors including salinity stress. Salt stress can affect ginseng growth and increase stress accumulation (Peng et al., 2007). Being that ginseng is a medicinal plant, decreases in growth rate can affect the nutritive value of the root and result in poor market value. The use of rhizobacteria has been well documented to promote plant growth as well as to alleviate various forms of abiotic stress including salinity. Application of PGPB for agricultural benefits has increased worldwide (Sukweenadhi et al., 2015). Microbial inoculants have considerable potential for use in agricultural biotechnology, based on these beneficial plant-microbe interactions. Salt stress in plants is a cumulative effect of osmotic and ionic stress that has a negative impact on plant growth and production. Depending on the plant species and soil environment, plant salt stress varies. In ginseng, we first analyzed the dose-dependent effect of salinity and found that 300 mM NaCl is the optimum level to induce salinity (Sukweenadhi et al., 2015). We also performed *in vitro* screening of several rhizobacteria isolated from ginseng soil and found that *Paenibacillus yonginensis* DCY84^T^ possessed the highest plant growth-promoting properties. Our previous findings also suggested that *P. yonginensis* DCY84^T^ inoculated in *Arabidopsis* under salt, drought and aluminum treatment led the highest resistance by activating several stress response genes. Based on the previous study, *P. yonginensis* DCY84^T^ is a promising candidate for application in crop cultivation. Therefore, in our present study we studied DCY84^T^'s role in ginseng growth by inoculating plants with or without salt stress conditions. We determined the ability of DCY84^T^ to improve salt tolerance in 2-year-old ginseng seedlings by measuring ROS including MDA, H_2_O_2_, chlorophyll, carotenoid content, sugars, antioxidant enzymes, proline, TSS and ion accumulation with or without salt exposure. We also identified changes in the expression of several genes involved in the salt stress tolerance mechanism.

The inoculation of bacteria on crop fields can be beneficial or harmful. We hence optimized the use of DCY84^T^ rhizobacteria as a microbial inoculant. The *in vitro* and *in vivo* assays indicated that 10 min dipping in DCY84^T^ at 10^8^–10^10^ CFU/ml is sufficient to alleviate salinity stress in ginseng. When salinity levels increase, water absorption is greatly reduced, aggravating water stress conditions (osmotic stress) in plants. Increasing the respiration rate is necessary for photosynthesis recovery during periods of water stress (Kirschbaum, 1988). From the total amount of carbon assimilated by photosynthesis, more than half is lost through the respiratory process needed for maintenance and plant growth, but this balance might change during water stress. The *RbohD* gene is involved in rapid long-distance signaling, which plays a role in cell-to-cell communication during plant response to pests, wounding, or extreme environmental conditions. Its signal propagation was induced by ROS accumulation (Møller, 2001). Similar to *RbohD, FER* also plays a role in cell-to-cell communication, as reported by Cheung and Wu. We found that stressed ginseng had a high oxidative respiration rate, as shown by high transcription levels of *PgRbohD* and *PgFER*. However, *PgFER* signaling was not as rapid as *PgRbohD*, as shown by the late upregulation of its transcription level. We found that ginseng under salt stress exhibited increased ROS accumulation by upregulating *PgRhoD* and *PgFER* expression. Any later effect of ROS can trigger accumulation of MDA and H_2_O_2_. In some C3 plants, limited CO_2_ fixation due to environmental stress can activate the photorespiratory pathway, generating H_2_O_2_ in peroxisomes through the enzymatic activity of glycolate oxidase (Davey et al., 2005). A recent report suggested that the salt-induced burst in ABA biosynthesis is correlated with carotenoid biosynthesis (Ruiz-Sola et al., 2014). The plants inoculated with DCY84^T^ exhibited high proline and decreased malondialdehyde and H_2_O_2_ levels under salt stress conditions when compared with un-inoculated salt stressed plants. Plants treated with strain DCY84 may be less sensitive to proline-mediated feedback inhibition via crosstalk with proline biosynthesis. In a previous report, H_2_O_2_ produced by NADPH oxidase was able to induce proline accumulation during salt stress in *A. thaliana* (Rejeb et al., 2015).

The increase of MDA content under salt stress is the result of membrane damage due to oxidative stress. The reduced MDA content during salt stress in DCY84^T^-inoculated plants was consistent with earlier findings (Bharti et al., 2014). Excessive accumulation of ROS can be harmful for plants in many ways, including degradation of chlorophyll, carotenoids and enzymes. Long-term salinity stress produced severe symptoms in the ginseng seedlings: withered, dried leaves as well as yellowing, shrunken roots. The morphological changes in the leaves were correlated with decreased chlorophyll and carotenoid content, while DAB and NBT staining indicated that ROS production and generated ROS might be responsible for various biochemical changes. When salt-stressed seedlings were inoculated with DCY84^T^, activation of *PgAAO3* occurred, which could result in ABA accumulation. The plant hormone ABA, a stress signal, plays a crucial role in regulating plant responses to water stress, not only by inducing stomatal closure (Lawson and Blatt, 2014) but also by enhancing tolerance to later oxidative stress by inducing the antioxidant defense system (Cutler and Krochko, 1999). ABA biosynthesis can use two possible pathways: a direct pathway from farnesyl pyrophosphate or an indirect pathway through the cleavage of a carotenoid precursor named xanthoxin (Burden and Taylor, 1976). It has also been proposed that ABA and xanthoxin arise from the breakdown of carotenoids in plant leaves (Volkov, 2015). High transcription levels of ABA-synthesis-related genes and antioxidant defense genes matched with high antioxidant enzyme activity explains how the primed ginseng seedlings can bear a long period of salinity stress. High levels of ABA induce antioxidant defense genes, such as *SOD, GPX, APX*, and *CAT*, that can scavenge ROS during later salinity-related damage from osmotic or ionic stress (Bharti et al., 2016). Chlorophyll and carotenoid breakdown often occurred in the stressed ginseng seedlings, which might have triggered ABA production and the antioxidant defense system (only APX and CAT). However, the amount of antioxidant enzymes was very low compared to that in the primed ginseng seedlings, which explains the tolerance of the primed seedlings (but not the stressed seedlings) during long-term salinity stress.

A later effect of salinity on living plants is ionic stress. Rising levels of salt in the cytoplasm, mostly sodium ions, cause toxic effects in plant cells. Ion fluxes, which control ion concentration, are essential for salinity tolerance. The best example is halophyte plants, which can grow at high salt concentrations (Platten et al., 2004). Halophytes can cope with high salt conditions using several strategies. For instance, they accumulate high sodium in their shoots, exclude sodium from their roots, localize salts into vacuoles, or excrete excess salt via salt glands. The role and proportion of each strategy depends on the plant type and habitat (Munns and Tester, 2008). However, most plant species are glycophytes, including ginseng. DCY84^T^ treatment of ginseng seedlings activated the transcription of several ion-pump-related genes responsible for maintaining ion fluxes that could be involved in preventing the toxic effects of ionic stress. As high sodium levels occurred, a low affinity Na^+^ transporter called HKT1 helped move Na^+^ into the cytosol (Laohavisit et al., 2013). Excess Ca^2+^ can protect plants from Na^+^ toxicity. Compelling evidence gathered over the years has shown that after the perception of salt stress, a Ca^2+^ spike generated in the cytoplasm of root cells activates a SOS signal transduction cascade to protect cells from damage due to excessive sodium ion accumulation (Quintero et al., 2002; Chinnusamy et al., 2005; Quan et al., 2007). It starts by activating the SOS3 calcium binding protein to bind and activate SOS2, a protein kinase family member (Zhu, 2000). In this study, we found very high expression levels of SOS3 and SOS2 in the seedlings primed with DCY84^T^ even before exposure to salinity stress, indicating that SOS signaling was somehow already idle. This SOS3-SOS2 complex was previously reported to bind with a Na^+^/H^+^ antiporter (Qiu et al., 2002; Chen et al., 2014). Our transcription analysis demonstrated that *SOS1* encodes a plasma membrane Na^+^/H^+^ antiporter that was upregulated during salinity stress (Shi et al., 2000; Quintero et al., 2011). On the other hand, its isoform (located on a vacuole regulated by *NHX1*) did not change significantly. These results indicate that ginseng cells tend to pump excess sodium ions out of cells, rather than store them in cell compartments. A similar tendency was recently reported in *Populus* (Martínez-Alcántara et al., 2015), but the reverse was reported for citrus (Peng et al., 2016) and upland cotton (Zhang et al., 2004). This pumping process was coupled with the electrochemical gradient generated by H^+^-ATPases. Apart from the SOS2-SOS3 complex, SOS2 can independently activate the pyridoxal kinase SOS4 to generate an active form of vitamin B6 (Shi et al., 2002), pyridoxal-5-phosphate (PLP), as reported (Rueschhoff et al., 2013). PLP might regulate ion channels or transporters in salt tolerance mechanisms (Shi and Zhu, 2002). Our finding of synergy in the high transcription levels of *SOS1, SOS2*, and *SOS4* confirms that previous report. Furthermore, we found that SOS4 indirectly affected the activation of the K^+^ channel AKT1 to maintain K^+^ levels inside the cytosol. In these ways, the primed ginseng seedlings coped with the ionic stress caused by sodium toxicity by pumping sodium out of cells rather than storing it inside compartments and maintaining potassium levels inside cells. The ion profile also revealed that primed ginseng plants had higher K^+^ and lower Na^+^ during salinity stress than stressed plants. A previous report on soybeans (Martínez-Alcántara et al., 2015) described the importance of maintaining the Na^+^/K^+^ ratio with regard to salt tolerance.

Apart from salt tolerance activity of DCY84^T^ we also analyzed growth-promoting activity. The strain DCY84^T^ produces 52.96 ± 1.85 μg/ml and 72.83 ± 2.86 μg/ml of indole-3-acetic acid without and with L-tryptophan, respectively. We also evaluated its growth-promoting activity by quantifying the expression pattern of root meristem genes such as *PgLAX, PgHB8*, and *PgPIN*. Upon priming of DCY84^T^, expression was dramatically increased in all three genes compared with salt-stressed seedlings, indicating that DCY84^T^ has plant growth-promoting characteristics. In addition, DCY84^T^ increased nutrient availability in salt-stressed seedlings by accumulating dehydrogenase, invertase and amylase.

In conclusion, DCY84^T^ increased nutrient availability (mostly sugar) and provided osmolytes to help ginseng plants tolerate short-term salinity stress (osmotic stress). During long-term salinity stress, activation of salt-defense-related genes such as ABA synthesis genes, ROS scavenging genes, ion-pump-related genes, and root meristem genes prevented both long- and short-term salinity stress. Moreover, DCY84^T^ exhibited growth-promoting properties even under salinity stress. Therefore, DCY84^T^ can strengthen plants as a microbial inoculant in ginseng fields affected by salinity stress.

## Author contributions

Yu-JK conceived the original screening and research plans. DY supervised the experiments. JS and SB performed most of the experiments. Ye-JK and SK performed quantitative real-time PCR. CL analyzed metabolite profiles. SB and JS wrote the manuscript and critically interpreted the data.

### Conflict of interest statement

The authors declare that the research was conducted in the absence of any commercial or financial relationships that could be construed as a potential conflict of interest.
