# Supplementary material for: A Growth-Promoting Bacteria, Paenibacillus yonginensis DCY84T Enhanced Salt Stress Tolerance by Activating Defense-Related Systems in Panax ginseng
Source: Front Plant Sci. 2018 Jul 23;9:813. doi: 10.3389/fpls.2018.00813 (PMC6065202; doi:10.3389/fpls.2018.00813)
Supplement: Supplementary file 1 [file Data_Sheet_1.docx]

**SUPPLEMENTARY INFORMATION**

**Supplementary Figure Legends**

**Supplementary Fig. S1.** Antifungal activity test of strain DCY84^T^ and other PGPB strains.

**Supplementary Fig. S2.** PCR results using a primer designed from 16S and 30S rRNA promoter of strain DCY84^T^.

**Supplementary Fig. S3.** Severity scale for the *in vitro* ginseng disc test.

**Supplementary Fig. S4.** Salinity tolerance limit of *P. ginseng* seedlings.

**Supplementary Table Legends**

**Supplementary Table S1** qRT-PCR primers used in this study

**Supplementary Table S2** *In vitro* screening of plant-growth promoting properties of DCY84^T^ and other isolated strains

**Supplementary Table S3** PGPB and pathogenic fungi growth check on various dual media

**Supplementary Table S4** Correlation of CFU/mL and OD value at 600 nm

**Supplementary Table S5** Correlation of OD value at 600 nm and Ct value

**Supplementary Table S6** Analysis of the soil sample (vermiculite:perlite:peat moss = 3:1:1)

**Supplementary Table S7** Promoter prediction value from the ginseng ORF database

**
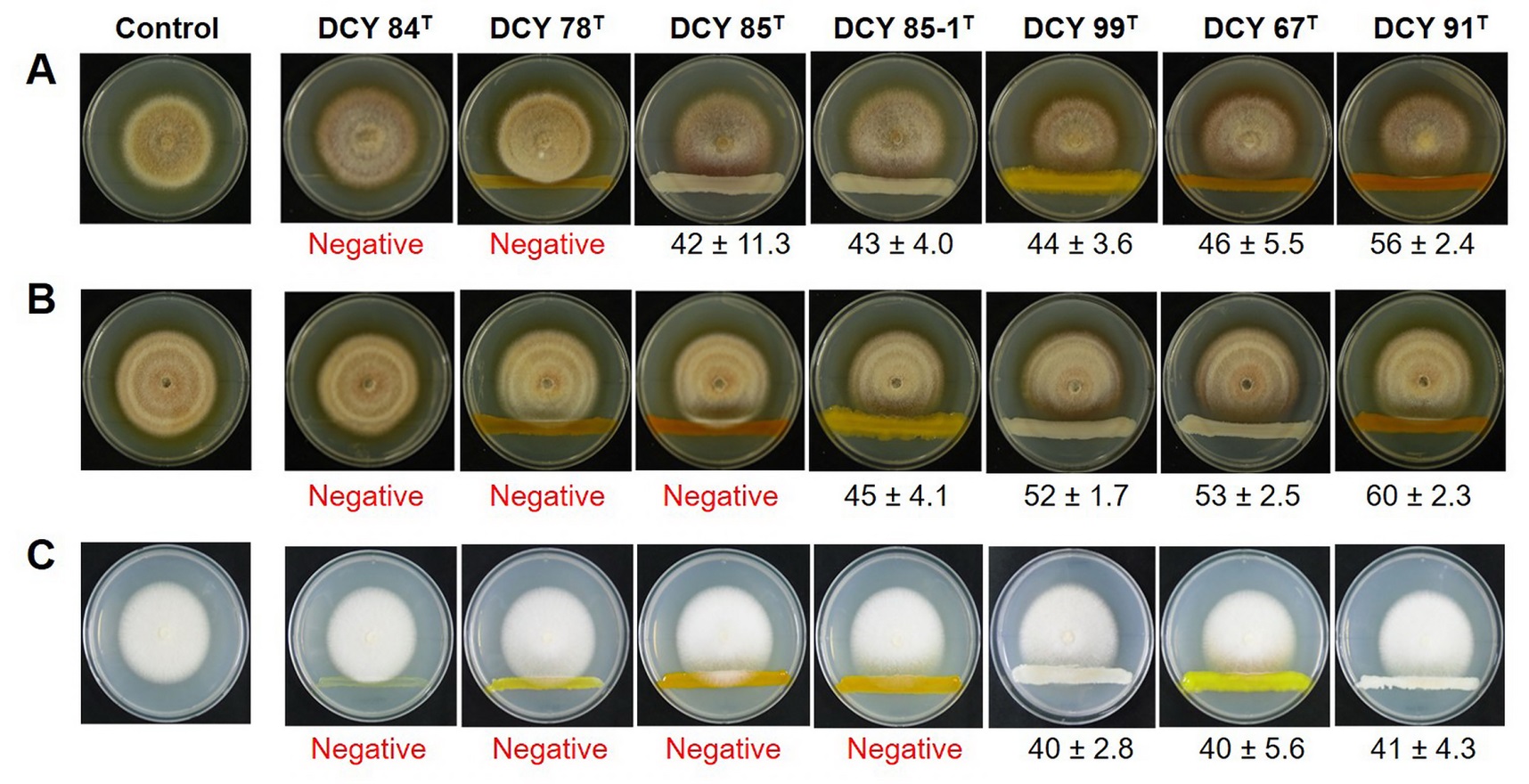
**

**Supplementary Figure S1. Antifungal activity test of strain DCY84^T^ and other PGPB strains.** The following PGPBs were tested for anti-fungal activity: (A) *Ilyonectria robusta* HB1; (B) *Ilyonectria robusta* HB4 and (C) *Fusarium solani* KACC44891^T^. We measured the suppression rate (% SR) by comparing the diameter of fungal mycelia on the plate (against bacterial streak on one side and no bacterial streak on the other side) with the control (only fungi). The standard error (SE) was calculated from three independent biological replications.

**
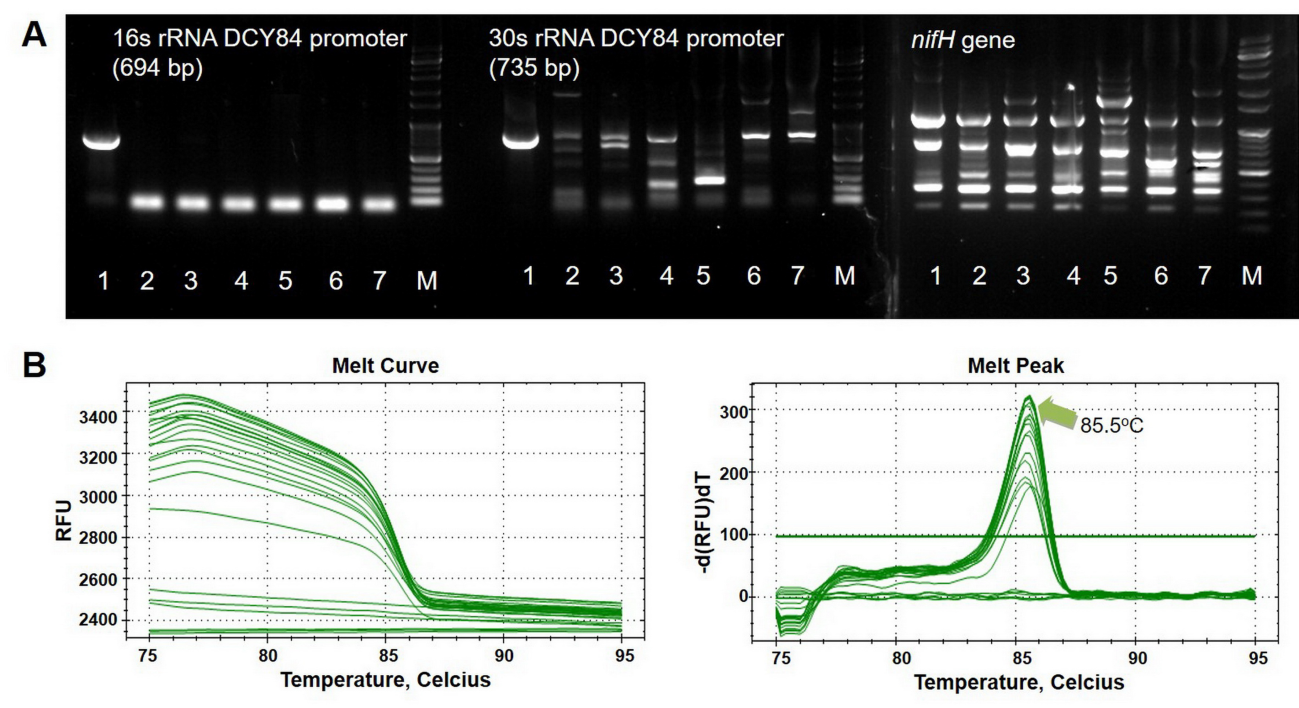
**

**Supplementary Figure S2. PCR results using the primer designed from 16S and 30S rRNA promoters of strain DCY84^T^.** (A) Seven different bacterial DNA were used as the template: 1, *P. yonginensis* DCY84^T^; 2, *P. phoenicis* NBRC 106274^T^; 3, *P. timonensis* KACC 11491^T^; 4, *P. barengoltzii* NBRC 106274^T^; 5, *Sphingomonas asaccharolytica* NBRC 15499^T^; 6, *S. panaciterrae* DCY91^T^; 7, *Burkholderia ginsengiterrae* DCY85-1^T^. We selected the *nifH* gene primer as the positive control for the PCR process. The primer for 16SrRNA was derived from the 694-bp promoter region of orf05875 and annotated as 16S rRNA methyltransferase, whereas the primer for 30SrRNA was derived from the 735-bp promoter region of orf03322 and annotated as 30S ribosomal protein s4. Both genes were obtained from the full genome of DCY84^T^. (B) We checked the specificity of the 16S RNA promoter region primer using qRT-PCR. Neither the curve nor the peak was detected in other bacterial samples or the water used as the template. Based on melting curve analysis, the strain DCY84^T^ marker derived from the 16S RNA promoter region has a melt curve temperature of 85.5ºC. The standard error (SE) was calculated from three independent biological replications.


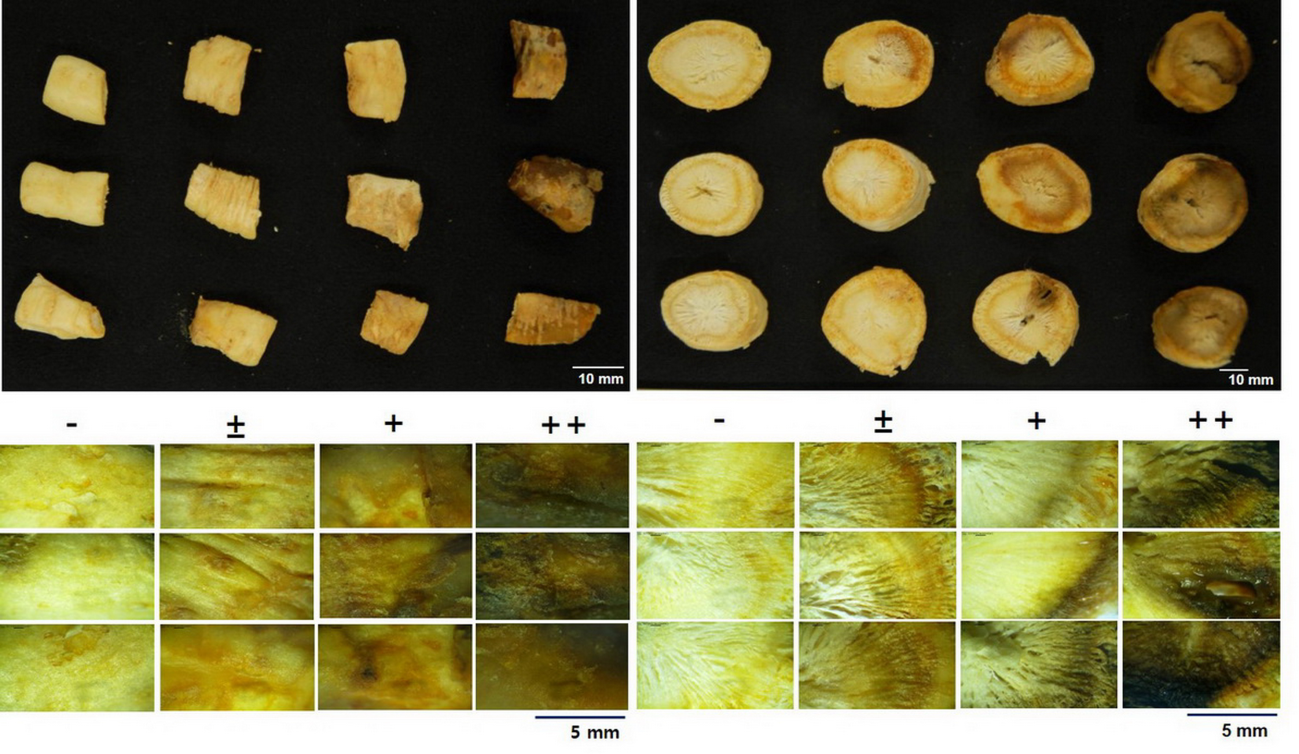


**Supplementary Figure S3. Severity scale of *in vitro* ginseng disc test.** Seven days post treatment, some root rot symptoms appeared, which we grouped into a 4-level severity scale; -, no symptoms; ±, yellowish discoloration or small lesion formation; +, mild brown rot, some part of root tissue starts to become soft; and ++, severe brown rot, root tissue becomes soft. The symptoms we observed from both the epidermis and cortex of the ginseng discs. Scale bar indicates 5 mm.

**
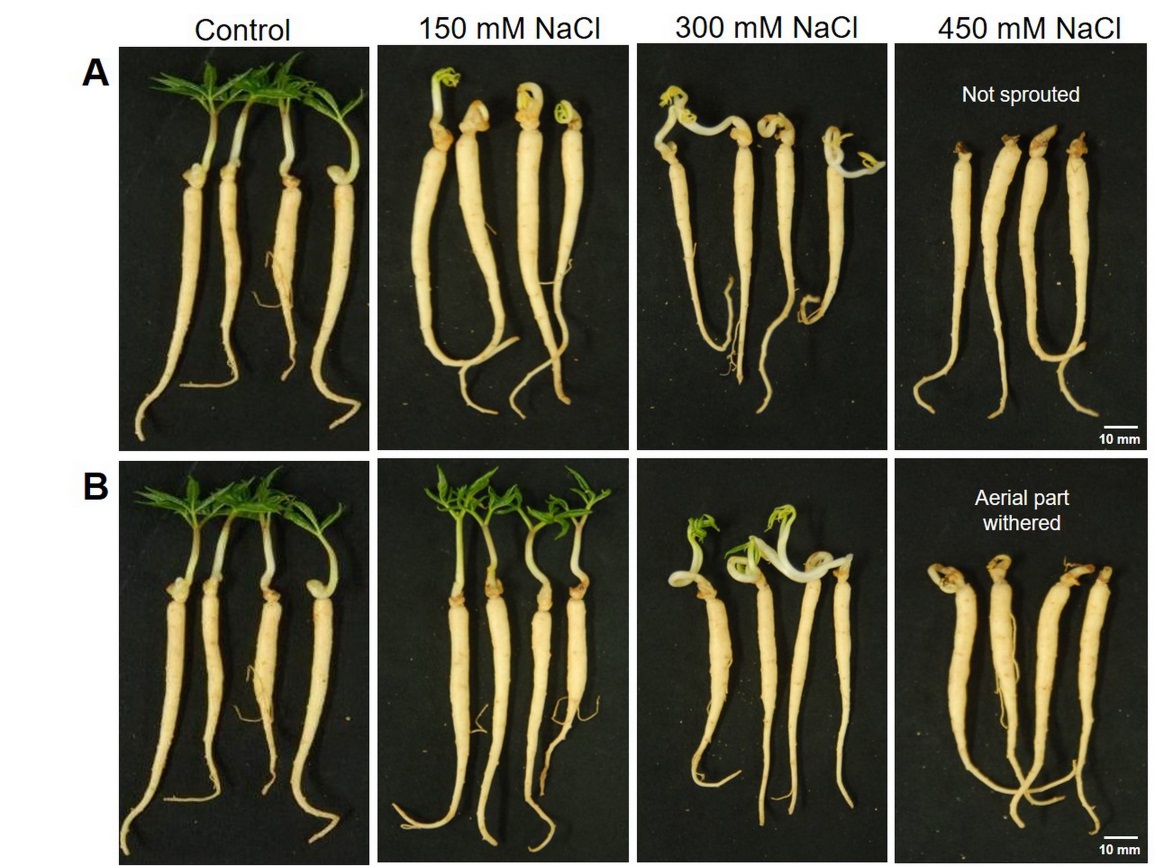
**

**Supplementary Figure S4. Salinity tolerance limit of *P. ginseng* seedlings.** Two-year-old *P. ginseng* plants were exposed to salinity stress at various NaCl concentrations (A) before and (B) after sprouting. Morphological appearance was observed after 10 days of cultivation.

**Supplementary Table S1.** qRT-PCR primers used in this study

| **Gene target** | **Primer sequence 5' - 3'** | | **Annealing temp. (ºC)** |
| --- | --- | --- | --- |
| ***Panax ginseng* Meyer** |  |  |  |
| *PgLAX3* | F: | GACAACTTACACTGCATGGT | 56 |
|  | R: | CGCATGCATTATCTCCACTG |  |
| *PgLATD* | F: | TTTTGGGATGGCTCTGGCT | 62 |
|  | R: | AGAGCCATAAGCCCAACAAC |  |
| *PgHB8* | F: | ACTCGGATGCTGTGATCTG | 58 |
|  | R: | TTGCAGAATTTGTGGGAACA |  |
| ***PgSOS1*** | F: | TTTATGAGTTGGCCTCAACA | 58 |
|  | R: | TTTACTTGCGGGAAATCTGG |  |
| ***PgSOS2*** | F: | CAGGCTATCTCCCATTTA | 56 |
|  | R: | CCGGAACCATGGATCATCT |  |
| *PgSOS3* | F: | TTTGCACTCTTCAGGAACAG | 62 |
|  | R: | TTTGCCAGATACTGGTGCAT |  |
| *PgSOS4* | F: | CATTCTTCATGCAGCTGG | 62 |
|  | R: | CCCGTGAAATATGCAGGAAT |  |
| *PgNHX1* | F: | ACAAGAGGAGGCCACACT | 56 |
|  | R: | TTGGGAGTGACTGGTTCAGA |  |
| *PgAKT1* | F: | GATGGGAGTGTACCACTATG | 56 |
|  | R: | GGCAGTCGATCCATTGTTTT |  |
| *PgRbohD* | F: | GGCTTGCTTCCTCGTACAC | 62 |
|  | R: | AAGCCGGGAATCAAAACTCT |  |
| ***PgAAO3*** | F: | TGTTGGCAATCTTGGGAGAAT | 62 |
|  | R: | TCCCTCTTGGAGTCCATTTG |  |
| *PgABI1* | F: | TTACTGGTGACCGGATTA | 56 |
|  | R: | ACAGCGTTAGCCAGGATTTG |  |
| *PgTHE1* | F: | TCACCGAGATGTGAAGAC | 56 |
|  | R: | AAGCTGGTCTTGTGCAGAGAA |  |
| *PgFER* | F: | GAGCAAGTTAGTTTGGCTGA | 62 |
|  | R: | CTCGAGGTTCCACAGGACAT |  |
| ***PgP5CS1*** | F: | TTTTGAGTCGCGTCCAGAA | 62 |
|  | R: | GCCAACACTTTTCGGGATAG |  |
| ***PgMYB1*** | F: | GCTCGTTGTGCTGGACTGA | 62 |
|  | R: | GGGCAAGTGCTGTGCTATTT |  |
| *PgPAO4* | F: | CTTTTGGCCAAATGTGGAAC | 62 |
|  | R: | CATTTTCCTGAGCTGCAACA |  |
| *PgSOD* | F: | ACCCGGACTTCATGGTTTTC | 56 |
|  | R: | CCCTTCCAATGATGGAACCT |  |
| *PgGPX* | F: | GTCAAGGATGCTAGAGGGAATG | 62 |
|  | R: | GTCCCTGGCTCCTGTGCT |  |
| ***PgCAT*** | F: | GAGCGTGGAAGTCCTGAGAC | 62 |
|  | R: | AATGTGAGACTTCGGGTTGG |  |
| ***PgAPX*** | F: | ATGGGAAAGTGCTACCCG | 60 |
|  | R: | TGAACATGCTCACCCTTAATTCT |  |
| *PgCYP* | F: | CAGGCAAAGAAAAAGTCAAGTG | 56 |
| (Liu *et al.*, 2014) | R: | AAAGAGACCCATTACAATACGC |  |

**Supplementary Table S2.** *In vitro* screening of the plant-growth promoting properties of DCY84^T^ and other isolated strains

| **Bacteria name** | **IAA concentration (µg/mL)** | | **SP^a^** | **PS^b^** | **Isolation source** | |
| --- | --- | --- | --- | --- | --- | --- |
|  | **Without L-tryptophan** | **With L-tryptophan** |  |  |  |  |
| DCY84^T^ (previously named PGPB8) | 52.9 ± 1.85 | 72.8 ± 2.26 | + | + | Yongin forest soil | |
| *P. barengoltzii* | 50.2 ± 0.45 | 70.8 ± 1.91 | + | + |  | |
| *P. phoenicis* | 12.3 ± 0.73 | 32.8 ± 4.57 | + | - |  | |
| *P. timonensis* | 29.5 ± 1.05 | 56.9 ± 3.55 | + | - |  | |
| *P. polymyxa* | 80.1 ± 0.69 | 85.6 ± 5.70 | + | + |  | |
| *Paenibacillus ginsengiterrae* DCY89^T^ | 9.5 ± 1.89 | 13.2 ± 0.11 | - | - | Suwon ginseng soil | |
| *P. cellulosilyticus* | 8.8 ± 2.41 | 49.8 ± 4.25 | + | - |  | |
| *P. curdlanolyticus* | 7.0 ± 1.41 | 23.2 ± 2.84 | + | + |  | |
| *P. xylaniclasticus* | 5.3 ± 1.05 | 7.0 ± 0.94 | - | + |  | |
| *P. kobensis* | 0.2 ± 0.05 | 0.9 ± 0.08 | + | + |  | |
| *Paenibacillus panaciterrae* DCY95^T^ | 6.9 ± 1.54 | 6.6 ± 2.24 | + | - | Quang Nam ginseng soil (Vietnam) | |
| *P. taihuensis* | 7.4 ± 1.68 | 2.5 ± 1.35 | + | - |  | |
| *P. sacheonensis* | 7.1 ± 1.89 | 2.1 ± 0.74 | + | + |  | |
| *Paenibacillus puernese* DCY97^T^ | 10.6 ± 2.36 | 15.0 ± 2.11 | + | - | Puer tea (China) | |
| *P. oceanisediminis* | 8.7 ± 1.87 | 9.2 ± 0.59 | w | w |  | |
| *Paenibacillus kyungheensis* DCY88^T^ | 8.3 ± 2.68 | 13.8 ± 3.65 | w | + | Magnolia flower (Korea) | |
| *P. hordei* | 5.3 ± 1.26 | 7.3 ± 2.65 | + | + |  | |
| *P. peoriae* | 7.3 ± 2.12 | 7.5 ± 3.12 | w | w |  | |
| *P. illinoisensis* | 9.2 ± 2.36 | 9.4 ± 2.65 | w | w |  | |
| *P. hunanensis* | 8.1 ± 2.57 | 8.2 ± 3.15 | + | - |  | |
| *P. kribensis* | 18.7 ± 4.68 | 19.5 ± 6.15 | + | + |  | |
| *Sphingomonas panaciterrae* DCY91^T^ | 9.6 ± 1.73 | 33.7 ± 4.66 | w | + | Suwon ginseng soil | |
| *S. mucossisima* | 2.9 ± 0.31 | 11.2 ± 1.96 | - | - |  | |
| *S. dokdonensis* | 24.5 ± 2.76 | 39.1 ± 3.72 | - | + |  | |
| *S. xinjiangensis* | 5.4 ± 0.15 | 14.1 ± 2.55 | - | w |  | |
| +, positive results; -, negative results; w, weakly positive results  ^a^Siderophore production; ^b^Phosphate solubilization | | | | | |  |

**Supplementary Table S2** (continued)

| **Bacteria name** | **IAA concentration (µg/mL)** | | **SP^a^** | **PS^b^** | **Isolation source** |
| --- | --- | --- | --- | --- | --- |
|  | **Without L-tryptophan** | **With L-tryptophan** |  |  |  |
| *Burkholderia ginsengiterrae* DCY85^T^ | 5.6 ± 0.98 | 44.3 ± 10.05 | + | w | Gochang diseased ginseng soil |
| *Burkholderia panaciterrae* DCY85-1^T^ | 4.2 ± 0.56 | 46.3 ± 2.72 | + | w |  |
| *B. bryophila* | 3.6 ± 0.22 | 8.8 ± 0.15 | + | + |  |
| *B. megapolitana* | 8.9 ± 0.15 | 9.8 ± 0.36 | - | - |  |
| *B. fungorum* | 3.3 ± 0.19 | 5.9 ± 0.28 | + | + |  |
| *B. caledonica* | 6.4 ± 0.36 | 7.1 ± 0.41 | + | + |  |
| *Sphingomonas panaciterrae* DCY91^T^ | 9.6 ± 1.73 | 33.7 ± 4.66 | w | + | Suwon ginseng soil |
| *S. mucossisima* | 2.9 ± 0.31 | 11.2 ± 1.96 | - | - |  |
| *S. dokdonensis* | 24.5 ± 2.76 | 39.1 ± 3.72 | - | + |  |
| *S. xinjiangensis* | 5.4 ± 0.15 | 14.1 ± 2.55 | - | w |  |
| *Sphingomonas panacis* DCY99^T^ | - | 22.4 ± 8.37 | - | + | Mountain ginseng soil (Hwacheon) |
| *S. asaccharolytica* | - | 18.6 ± 4.56 | - | - |  |
| *Duganella ginsengisoli* DCY83^T^ | 10.6 ± 2.51 | 12.9 ± 2.25 | + | + | Gwangju ginseng soil |
| *D. phyllosphaerae* | 12.9 ± 3.11 | 24.8 ± 4.65 | + | + |  |
| *D. radices* | 27.9 ± 5.26 | 37.9 ± 5.74 | + | - |  |
| *D. sacchari* | 10.3± 1.78 | 24.9 ± 3.69 | + | + |  |
| *D. zoogloeoides* | 5.4 ± 0.98 | 14.7 ± 3.32 | + | + |  |
| *Cupriavidus*  *yeoncheonense* DCY86^T^ | 8.2 ± 2.23 | 27.3 ± 4.11 | + | - | Yeoncheon ginseng soil |
| *C. laharis* | 15.7 ± 3.11 | 16.9 ± 3.45 | + | + |  |
| *C. numazuensis* | 0.2 ± 0.05 | 9.8 ± 2.13 | + | - |  |
| *C. pinatubonensis* | 3.5 ± 0.06 | 9.4 ± 2.11 | + | - |  |
| *C. basilensis* | 7.5 ± 0.09 | 14.1 ± 1.22 | + | + |  |
| *Humibacter ginsengiterrae* DCY60^T^ | 6.4 ± 1.25 | 10.90 ± 2.51 | - | + | Yeoncheon ginseng soil |
| *Humibacter ginsengisoli* DCY90^T^ | 9.0 ± 2.15 | 11.87 ± 2.24 | - | + | Gochang diseased ginseng soil |
| +, positive results; -, negative results; w, weakly positive results  ^a^Siderophore production; ^b^Phosphate solubilization | | | | | |

**Supplementary Table S2** (continued)

| **Bacteria name** | **IAA concentration (µg/mL)** | | **SP^a^** | **PS^b^** | **Isolation source** |
| --- | --- | --- | --- | --- | --- |
|  | **Without L-tryptophan** | **With L-tryptophan** |  |  |  |
| *Phycicoccus* DCY87^T^ | 23.3 ± 2.55 | 24.7 ± 2.62 | + | + | Yeoncheon ginseng soil |
| *P. ginsenoisidimutans* | 12.7 ± 1.98 | 14.1 ± 1.45 | - | + |  |
| *P. badiiscoriae* | 8.1 ± 0.98 | 15.1 ± 1.84 | + | + |  |
| *P. cremeus* | 12.8 ± 1.56 | 22.6 ± 2.36 | - | - |  |
| *P. dokdonensis* | 14.3 ± 2.21 | 15.7 ± 1.44 | - | - |  |
| *P. jejuensis* | 10.3 ± 0.98 | 11.5 ± 0.98 | + | + |  |
| *P. ochangensis* | 10.4 ± 0.65 | 19.5 ± 2.05 | + | + |  |
| *Paracoccus panacisoli* DCY94^T^ | - | 14.4 ± 0.58 | + | - | Vietnam ginseng soil |
| *P. sphaerophysae* | - | 40.7 ± 0.20 | + | - |  |
| *P. caeni* | - | 11.4 ± 0.10 | + | - |  |
| +, positive results; -, negative results; w, weakly positive results  ^a^Siderophore production; ^b^Phosphate solubilization | | | | | |

**Supplementary Table S3** PGPB and pathogenic fungi growth check on various dual media

| **PGPB Candidate** | **YM MEDIA** | | | **TY MEDIA** | | | **WA MEDIA** | | |
| --- | --- | --- | --- | --- | --- | --- | --- | --- | --- |
|  | Day 1 | Day 2 | Day 3 | Day 1 | Day 2 | Day 3 | Day 1 | Day 2 | Day 3 |
| *Paenibacillus* DCY84^T^ | + | ++ | ++ | + | ++ | +++ | + | ++ | +++ |
| *Sphingomonas* DCY91^T^ | + | +++ | +++ | +++ | +++ | +++ | +++ | +++ | +++ |
| *Sphingomonas* DCY99^T^ | + | +++ | +++ | + | ++ | +++ | + | ++ | +++ |
| *Burkholderia* DCY85^T^ | + | ++ | +++ | +++ | +++ | +++ | + | ++ | +++ |
| *Burkholderia* DCY85-1^T^ | + | +++ | +++ | +++ | +++ | +++ | +++ | +++ | +++ |
| *Epilithonimonas* DCY78^T^ | + | ++ | ++ | +++ | ++ | ++ | +++ | +++ | +++ |
| *Chryseobacterium* DCY98^T^ | + | +++ | +++ | +++ | +++ | +++ | +++ | +++ | +++ |
| *Chryseobacterium* DCY67^T^ | + | +++ | +++ | +++ | +++ | +++ | +++ | +++ | +++ |
| **Pathogenic Fungi** | **YM MEDIA** | | | **TY MEDIA** | | | **WA MEDIA** | | |
|  | Day 3 | Day 5 | Day 10 | Day 3 | Day 5 | Day 10 | Day 3 | Day 5 | Day 10 |
| *Ilyonectria robusta* HB1 | + | + | +++ | + | + | ++ | + | + | +++ |
| *Ilyonectria robusta* HB4 | + | + | +++ | + | + | ++ | + | + | +++ |
| *Fusarium solani* KACC44891^T^ | ++ | +++ | +++ | ++ | +++ | +++ | ++ | +++ | +++ |

+, weak growth; ++, good growth; +++, very good growth.

All PGPB candidate strains were activated from stock culture in TSA vials and incubated for 2 days at their optimum temperature (25ºC or 30ºC). All pathogenic fungi were activated via culture on PDA medium and incubation at 25ºC for 5 days. YM, yeast malt agar; TY, Tryptone yeast agar; WA, Waksman agar.

**Supplementary Table S4.** Correlation of CFU/mL and OD value at 600 nm

| **Inoculum concentration (CFU/mL)** | ***Paenibacillus yonginensis***  **DCY84^T^ OD_600nm_** | ***Paenibacillus polymyxa***  **KACC 10485^T^ OD_600nm_** |
| --- | --- | --- |
| 10^2^ CFU/mL | 0.11 ± 0.024 | 0.14 ± 0.022 |
| 10^4^ CFU/mL | 0.45 ± 0.033 | 0.37 ± 0.017 |
| 10^6^ CFU/mL | 0.87 ± 0.031 | 0.60 ± 0.028 |
| 10^8^ CFU/mL | 1.28 ± 0.025 | 0.82 ± 0.033 |
| 10^10^ CFU/mL | 1.73 ± 0.027 | 1.05 ± 0.024 |
| 10^12^ CFU/mL | 2.16 ± 0.035 | 1.28 ± 0.014 |

**Supplementary Table S5.** Correlation of OD value at 600 nm and Ct value

| **OD value** | **Ct value** | | | **Mean Ct** | **Predicted log (CFU/mL)*** | **Predicted CFU/mL**** |
| --- | --- | --- | --- | --- | --- | --- |
| 1.71 | 16.55 | 16.64 | 16.72 | 16.637 | 10.0 | ~1.1x10^10^ |
| 1.33 | 17.78 | 17.65 | 17.83 | 17.753 | 8.3 | ~1.9x10^8^ |
| 0.95 | 18.71 | 18.85 | 18.88 | 18.813 | 6.5 | ~3.2x10^6^ |
| 0.85 | 19.05 | 19.26 | 19.11 | 19.140 | 6.0 | ~1.1x10^6^ |
| 0.42 | 20.63 | 20.74 | 20.81 | 20.727 | 4.0 | ~1.1x10^4^ |
| 0.15 | 21.65 | 21.77 | 21.89 | 21.770 | 2.8 | ~6.2x10^2^ |

*Calculated using DCY84^T^ equation for OD and log (CFU/mL): y=4.6396x + 2.0996 (from the data shown in Supplementary Table S5)

**Calculated using 10^[log(CFU/mL)]

**Supplementary Table S6.** Analysis of the soil sample (vermiculite:perlite:peat moss = 3:1:1)

| Treatment to soil | pH | EC (dS/m) | Ex-cations (cmol/kg) | | | | Other chemicals (mg/kg) | | | | | | | Organic matter (g/kg) | Av. P_2_O_5_ (mg/kg) | Av. SiO_2_ (mg/kg) |
| --- | --- | --- | --- | --- | --- | --- | --- | --- | --- | --- | --- | --- | --- | --- | --- | --- |
|  |  |  | K | Ca | Mg | Na | Cl^-^ | SO_4_^2-^ | NO_3_-N | Fe | Mn | Zn | Al |  |  |  |
| NaCl 0 mM | 6.70 | 0.09 | 10.3 | 260 | 11.31 | 6.2 | 10.26 | ND | 26.22 | ND | ND | ND | ND | 8.584 | 60 | 35.962 |
| NaCl 150 mM | 6.54 | 0.81 | 14.6 | 272 | 14.56 | 330 | 14.59 | ND | 27.73 | ND | ND | ND | ND | 8.740 | 63 | 35.474 |
| NaCl 300 mM | 6.43 | 1.55 | 20.3 | 290 | 16.84 | 625 | 20.34 | ND | 28.66 | ND | ND | ND | ND | 8.744 | 66 | 35.621 |
| NaCl 450 mM | 6.30 | 2.71 | 34.7 | 353 | 19.79 | 1230 | 34.72 | ND | 38.54 | ND | ND | ND | ND | 8.608 | 70 | 35.690 |

ND, no data.

**Supplementary Table S7.** Promoter prediction value from the ginseng ORF database

| **ORF** | **Annotation** | **Promoter prediction value** | | | | |
| --- | --- | --- | --- | --- | --- | --- |
|  |  | **-200 bp** | **-300 bp** | **-400 bp** | **-500 bp** | **> -500 bp** |
| orf01463_contig.1.cir | 16s rrna processing protein | 0 | 0 | 0 | 0 | 0 |
| orf03267_contig.1.cir* | 16s rrna methyltransferase | 0 | 0 | 0 | 0.509 | 0 |
| orf03493_contig.1.cir | 16s rrna methyltransferase | 0 | 0 | 0 | 0 | 0 |
| orf03561_contig.1.cir | 16s rrna maturation rnase | 0 | 0 | 0 | 0 | 0 |
| orf03592_contig.1.cir | 16s rrna methyltransferase | 0 | 0 | 0 | 0 | 0 |
| orf00809_contig.1.cir | 23s rrna methyltransferase | 0 | 0 | 0 | 0 | 0 |
| orf03265_contig.1.cir | 23s rrna (adenine -c ) - methyltransferase | 0 | 0 | 0 | 0 | 0 |
| orf06218_contig.1.cir* | 23s rrna (adenine -c ) - methyltransferase | 0 | 0 | 0 | 0 | 0.613 |
| orf01461_contig.1.cir | 30s ribosomal protein s16 | 0 | 0 | 0 | 0 | 0.606 |
| orf01573_contig.1.cir | 30s ribosomal protein s2 | 0 | 0 | 0 | 0 | 0.526 |
| orf01599_contig.1.cir | 30s ribosomal protein s15 | 0 | 0 | 0 | 0 | 0.675 |
| orf02271_contig.1.cir | 30s ribosomal protein s14 | 0 | 0 | 0 | 0.546 | 0.713 |
| orf03322_contig.1.cir* | 30s ribosomal protein s4 | 0 | 0 | 0 | 0 | 1.322 |
| orf03626_contig.1.cir | 30s ribosomal protein s20 | 0 | 0 | 0 | 0 | 0.533 |
| orf00834_contig.1.cir | 50s ribosomal protein l35 | 0 | 0 | 0 | 0 | 0.583 |
| orf00836_contig.1.cir | 50s ribosomal protein l20 | 0 | 0 | 0 | 0 | 0.583 |
| orf01176_contig.1.cir | 50s ribosomal protein l31 type b | 0 | 0 | 0 | 0 | 0.594 |
| orf01592_contig.1.cir | 50s ribosomal protein l7ae | 0 | 0 | 0 | 0 | 0.558 |
| orf03597_contig.1.cir | 50s ribosomal protein l11 methyltransferase | 0 | 0 | 0 | 0 | 0.565 |
| orf04027_contig.1.cir | 50s ribosomal protein l25 | 0 | 0 | 0 | 0 | 0.564 |
| orf04188_contig.1.cir | 50s ribosomal protein l27 | 0 | 0 | 0 | 0 | 0.516 |
| orf04485_contig.1.cir | 50s ribosomal protein l21 | 0 | 0 | 0 | 0 | 0.545 |
| orf05063_contig.1.cir | 50s ribosomal protein l13 | 0 | 0 | 0 | 0 | 0.637 |
| orf05067_contig.1.cir | 50s ribosomal protein l17 | 0 | 0 | 0 | 0 | 0.57 |
| orf05079_contig.1.cir | 50s ribosomal protein l15 | 0 | 0 | 0 | 0 | 0.561 |
| orf05082_contig.1.cir | 50s ribosomal protein l18 | 0 | 0 | 0 | 0 | 0.512 |
| orf05868_contig.1.cir* | 50s ribosomal protein l34 | 0 | 0 | 0 | 0 | 1.079 |
| orf05924_contig.1.cir | 50s ribosomal protein l9 | 0 | 0 | 0 | 0 | 0.507 |

*The sequence upfront the ORF was used to design the specific genetic marker (primer) candidates for DCY84^T^

Promoter 2.0 software predicted the promoter region from the submitted sequence based on promoter prediction values:

<0.5 means ignored; 0.5–0.8 means marginal prediction; 0.8–1.0 means medium likely prediction, and >1.0 means highly likely prediction.
